# T-cell senescence contributes to abnormal glucose homeostasis in humans and mice

**DOI:** 10.1038/s41419-019-1494-4

**Published:** 2019-03-13

**Authors:** Hyon-Seung Yi, So Yeon Kim, Jung Tae Kim, Young-Sun Lee, Ji Sun Moon, Mingyo Kim, Yea Eun Kang, Kyong Hye Joung, Ju Hee Lee, Hyun Jin Kim, Kwangsik Chun, Minho Shong, Bon Jeong Ku

**Affiliations:** 1 Research Center for Endocrine and Metabolic Diseases, Chungnam National University Hospital, Chungnam National University School of Medicine, Daejeon, 35015 Republic of Korea; 2 Department of Internal Medicine, Chungnam National University School of Medicine, Daejeon, 35015 Republic of Korea; 3 Laboratory of Liver Research, Biomedical Science and Engineering Interdisciplinary Program, Korean Advanced Institute of Science and Technology, Daejeon, 34141 Republic of Korea; 4 Division of Digestive and Liver Diseases, Department of Medicine, Cedars-Sinai Medical Center, Los Angeles, CA 90048 USA; 5 Department of Medical Science, Chungnam National University School of Medicine, 266 Munhwaro, Daejeon, 35015 Republic of Korea; 6 Department of Internal Medicine, Korea University College of Medicine, Seoul, 08308 Republic of Korea; 7 Division of Rheumatology, Department of Internal Medicine, Gyeongsang National University School of Medicine, 79, Gangnam-ro, Jinju, Gyeongnam, 660-702 Republic of Korea; 8 Department of Surgery, Chungnam National University School of Medicine, Daejeon, 35015 Republic of Korea

## Abstract

Chronic inflammation is a driving force for the development of metabolic disease including diabetes and obesity. However, the functional characteristics of T-cell senescence in the abnormal glucose homeostasis are not fully understood. We studied the patients visiting a hospital for routine health check-ups, who were divided into two groups: normal controls and people with prediabetes. Gene expression profiling of peripheral blood mononuclear cells from normal controls and patients with type 2 diabetes was undertaken using microarray analysis. We also investigated the immunometabolic characteristics of peripheral and hepatic senescent T cells in the normal subjects and patients with prediabetes. Moreover, murine senescent T cells were tested functionally in the liver of normal or mice with metabolic deterioration caused by diet-induced obesity. Human senescent (CD28^−^CD57^+^) CD8^+^ T cells are increased in the development of diabetes and proinflammatory cytokines and cytotoxic molecules are highly expressed in senescent T cells from patients with prediabetes. Moreover, we demonstrate that patients with prediabetes have higher concentrations of reactive oxygen species (ROS) in their senescent CD8^+^ T cells via enhancing capacity to use glycolysis. These functional properties of senescent CD8^+^ T cells contribute to the impairment of hepatic insulin sensitivity in humans. Furthermore, we found an increase of hepatic senescent T cells in mouse models of aging and diet-induced obesity. Adoptive transfer of senescent CD8^+^ T cells also led to a significant deterioration in systemic abnormal glucose homeostasis, which is improved by ROS scavengers in mice. This study defines a new clinically relevant concept of T-cell senescence-mediated inflammatory responses in the pathophysiology of abnormal glucose homeostasis. We also found that T-cell senescence is associated with systemic inflammation and alters hepatic glucose homeostasis. The rational modulation of T-cell senescence would be a promising avenue for the treatment or prevention of diabetes.

## Introduction

Chronic inflammation is strongly associated with metabolic diseases, including diabetes and atherosclerosis^1,2^. Patients with insulin resistance are considered to be at greater risk of cardiovascular disease^3^. Proinflammatory cytokines, such as tumor necrosis factor-α (TNF-α), interleukin (IL)-1β, and IL-6, play essential roles in the pathogenesis of insulin resistance^4,5^. Moreover, patients with prediabetes show significantly lower insulin sensitivity and higher levels of inflammatory markers than metabolically normal individuals^6^. In addition, low-grade inflammation in prediabetes is thought to increase the risk of a cardiovascular event^7^.

Aging of the immune system also contributes to the development of chronic inflammation and has an important effect on metabolic disease and immunologic disorders in humans^8^. In addition, low-grade chronic inflammation is a driver of immunosenescence^9^. The chronic inflammatory environment that is a characteristic of metabolic diseases may also be induced by augmented secretion of proinflammatory cytokines, including TNF-α and IL-6, reactive oxygen species (ROS), and acute-phase reactants released from senescent immune cells. In human studies, several lines of evidence indicate that a senescent T-cell-mediated inflammatory response is associated with the pathogenesis of acute coronary syndrome and hypertension^10,11^. However, any relationship between the immunosenescence of T cells and abnormal glucose homeostasis remains to be elucidated.

The loss of the co-stimulatory molecule CD28 and the gain of CD57 expression are prominent markers of the aging immune system in human CD4^+^ or CD8^+^ T cells^12,13^. CD28 is downregulated after replicative senescence^14^, but loss or gain of CD28 is also associated with proinflammatory conditions and diseases^4,10,15–18^. These CD28^−^ T cells, which have shortened telomeres, show reductions in T-cell receptor diversity and cytotoxic capacity^12^. CD57^+^ T cells are proliferation incompetent in response to antigen-specific stimulation and susceptible to apoptosis upon T-cell activation^19,20^. Although these senescent T cells might contribute to the pathogenesis of immune disorders, the role of senescent T cells in metabolic diseases has yet to be determined.

In the present study, we investigate whether T-cell senescence contributes to the systemic inflammatory response in patients with prediabetes and mice with diet-induced obesity by immunologically characterizing senescent T cells. We also demonstrate that the presence of these senescent T cells is associated with hepatic inflammation and impaired glucose homeostasis in mice and humans. In summary, this study suggests the existence of an immunometabolic link between T-cell senescence and the pathophysiology of diabetes.

## Results

### Patients with type 2 diabetes exhibit systemic proinflammatory response

**Fig. 1:**
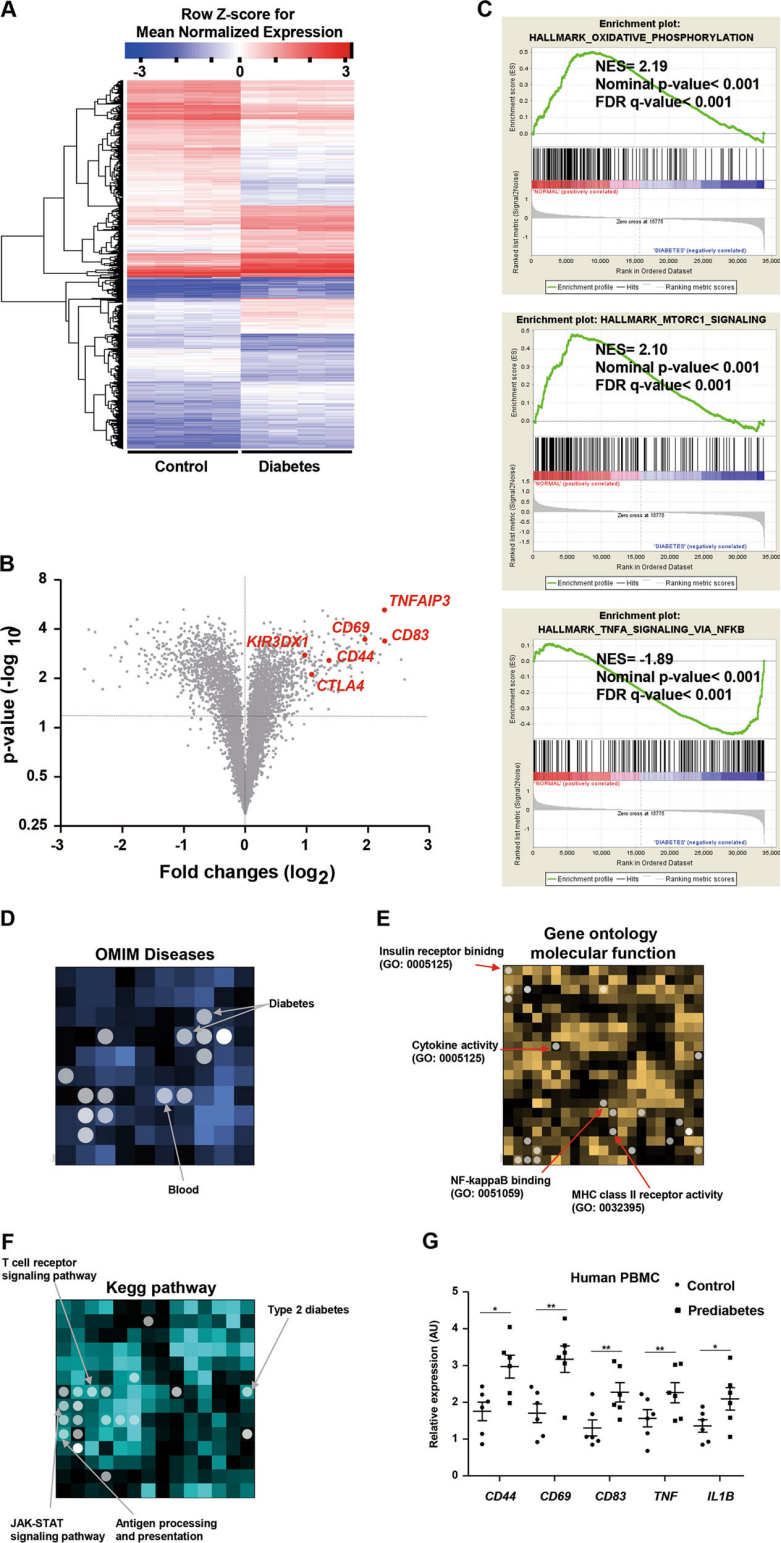
Identification of differentially expressed genes in PBMCs from normoglycemic controls and patients with prediabetes. **a** Hierarchical clustering and heatmap based on genes that were differentially expressed in PBMCs from patients with prediabetes (*n* = 4) (≥ 2-fold change in expression compared with normoglycemic controls (*n* = 4)). **b** Volcano plot based on the differential expression and significance of the differences in 44,547 Agilent gene probes. Red dots represent genes associated with T-cell activation and inflammation with *P* < 0.05 and fold change > 1 log2. **c** The diagram shows the result of Gene Set Enrichment Analysis, including the enrichment scores. **d–f** The analysis was performed using Network2Canvas. Genes that were significantly upregulated in the PBMCs of normal controls and patients with prediabetes were analyzed for gene-list enrichment with gene set libraries created from level 4 of the MGI mouse phenotype ontology using Network2Canvas. **g** PBMCs isolated from controls (*n* = 6) and patients (*n* = 6) with prediabetes were subjected to real-time PCR. Data are expressed as mean ± SEM. *P < 0.05 and **P < 0.01 compared with the corresponding controls

We compared dendrograms and cluster heatmap visualizations created using our heuristics with the default heuristic in R and seriation-based leaf ordering methods (Fig. 1a). The expression of 1324 genes differed between peripheral blood mononuclear cells (PBMCs) from drug-naive patients with type 2 diabetes and those from controls (Fig. 1a). We then found that the 10 representative terms Gene Ontology Biological Process and Cellular Component and Molecular Function were enriched in PBMCs from drug-naive patients with type 2 diabetes (Supplementary Fig. 1a–c). Interestingly, genes associated with the immune response, the defense response and the inflammatory response were enriched in PBMCs from the patients with type 2 diabetes (Fig. 1b). Consistent with this, gene set enrichment analysis revealed a strong correlation of genes upregulated in PBMCs from drug-naive patients with type 2 diabetes with a gene set that identifies TNF-α signaling via nuclear factor kappa B (Fig. 1c). Conversely, a gene set involved in oxidative phosphorylation and mTORC1 signaling was enriched in normal controls compared with patients with diabetes (Fig. 1c). Furthermore, enrichment analysis with Network2Canvas revealed differing expression of signaling molecules implicated in diabetes and the immune system between the two groups (Fig. 1d–f and Supplementary Fig. 1d).

### Prediabetes is associated with increased inflammation in humans

Patients with prediabetes also exhibit higher levels of inflammatory markers and are more susceptible to cardiovascular diseases^21^. However, it remains unclear what kind of immune cells contribute to the development of prediabetes. Thus, the study for the immune cells in patients with prediabetes possibly contributes to elucidating pathogenesis of overt diabetes. In this study, the 80 participants included in the final analysis were classified into two groups: normal controls and patients with prediabetes. The demographics and baseline characteristics of both the patients with prediabetes and the control subjects with normal glucose tolerance are summarized in Table 1. The expression of genes involved in human T-cell activation, including *CD44* and *CD69*, as well as proinflammatory cytokines, was significantly higher in patients with prediabetes (Fig. 1g). As shown in Supplementary Fig. 2, the patients with prediabetes showed higher serum levels of TNF-α than controls with normoglycemia. Serum IFN-γ and IL-1β also showed a tendency toward increase in the patients with prediabetes compared with normal controls (Supplementary Fig. 2a–c). These findings suggest that prediabetes is associated with systemic proinflammatory response.

**Table 1:** Demographics and baseline characteristics of both patients with prediabetes and normoglycemic control subjects

Variables	Control (*n* = 40)	Prediabetes (*n* = 40)	*P* value
Age, y	51.4 ± 12.1	54.8 ± 9.7	0.11
Male, *n* (%)	12 (30.0%)	12 (30.0%)	…
Height, cm	159.6 ± 7.3	159.1 ± 6.5	0.374
Weight, kg	59.4 ± 11.7	62.7 ± 11.1	0.134
BMI, kg/m^2^	23.3 ± 3.5	24.6 ± 3.9	0.074
Current smoking, *n* (%)	3 (7.50%)	3 (7.50%)	…
SBP, mm Hg	123.3 ± 15.3	126.7 ± 13.8	0.279
DBP, mm Hg	75.9 ± 11.1	76.6 ± 9.0	0.468
Hemoglobin, g/dL	13.3 ± 0.7	13.3 ± 1.1	0.143
HbA1c, %	5.3 ± 0.2	5.9 ± 0.3	<0.001
FBG, mg/dL	91.0 ± 5.6	100.2 ± 5.4	<0.001
PP2, mg/dL	100.1 ± 20.4	148.5 ± 28.3	0.002
Fasting insulin, μIU/mL	8.0 ± 3.1	9.5 ± 6.5	0.25
PP2 insulin, μIU/mL	31.1 ± 23.4	59.7 ± 62.6	0.045
Fasting c-peptide, pmol/mL	0.6 ± 0.4	0.7 ± 0.5	0.284
PP2 c-peptide, pmol/mL	2.6 ± 1.4	3.3 ± 1.7	0.073
HOMA-IR	1.8 ± 0.8	2.4 ± 1.7	0.127
Insulin (PP2/fasting)	3.6 ± 1.7	5.6 ± 3.2	0.009
c-peptide (PP2/fasting)	4.2 ± 1.3	5.0 ± 1.7	0.048
Total cholesterol, mg/dL	187.0 ± 36.8	197.3 ± 42.1	0.225
Triglyceride, mg/dL	123.0 ± 35.4	122.5 ± 36.6	0.352
HDL-C, mg/dL	64.5 ± 16.5	55.1 ± 12.7	0.022
LDL-C, mg/dL	81.2 ± 57.3	137.8 ± 80.9	0.003
Aspartate transaminase, IU/L	23.4 ± 20.9	23.3 ± 14.8	0.474
Alanine transaminase, IU/L	24.7 ± 32.4	25.4 ± 24.8	0.484
Creatinine, mg/dL	0.7 ± 0.1	0.7 ± 0.1	0.423
hsCRP, mg/dL	0.9 ± 0.8	1.5 ± 2.0	0.085

*BMI* body mass index *SBP*, systolic blood pressure, *DBP* diastolic blood pressure, *HbA1c* glycated hemoglobin, *FBG* fasting blood glucose, *PP2* post-prandial 2 h blood glucose, HOMA-IR homeostasis model assessment-estimated insulin resistance, *HDL-C* high-density lipoprotein cholesterol, *LDL-C* low-density lipoprotein cholesterol, *hsCRP* high-sensitivity C-reactive protein

### Senescent CD8^+^ T cells are more numerous in patients with prediabetes in humans

**Fig. 2:**
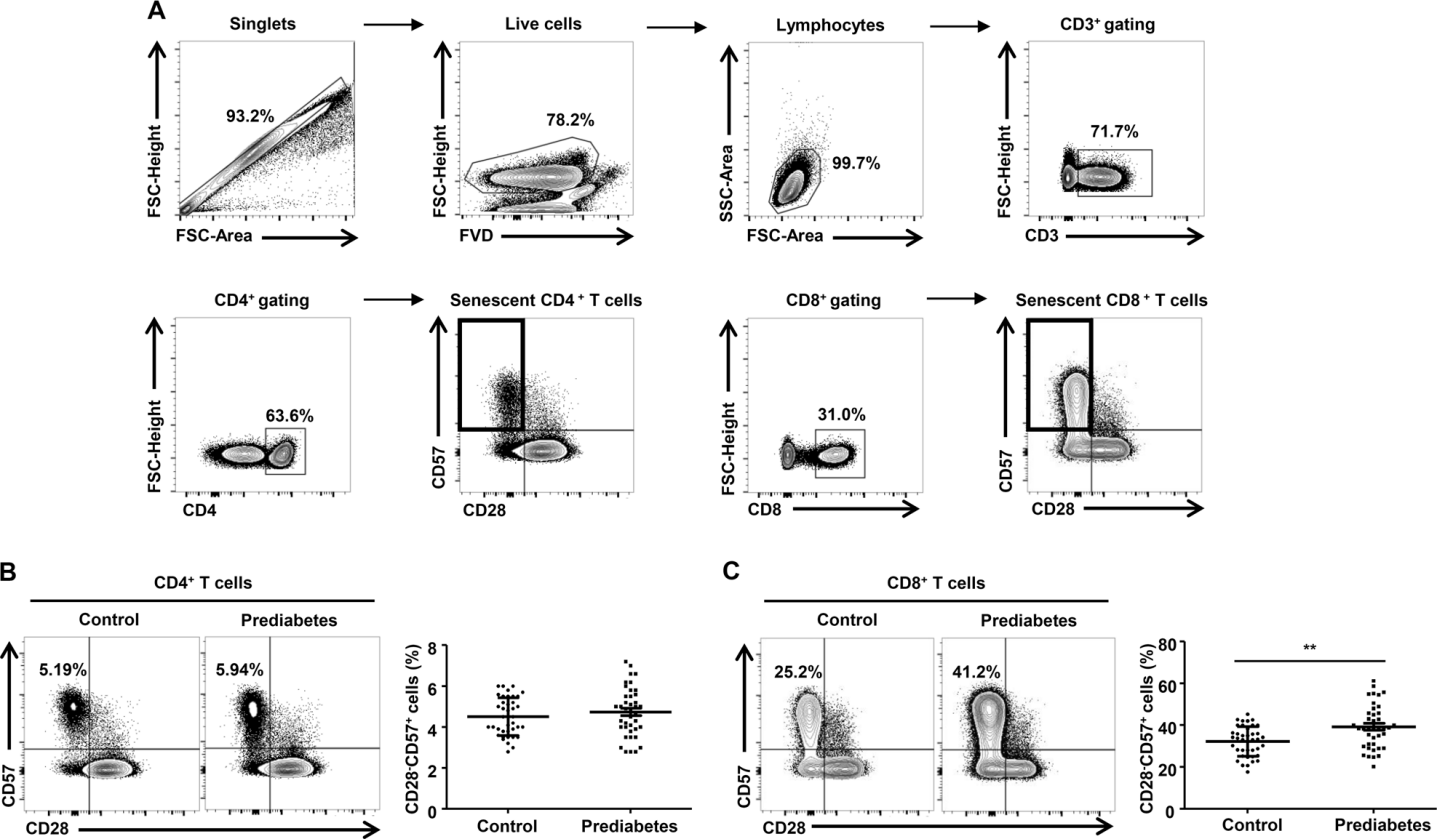
Population size of CD28^−^CD57^+^ T cells from normoglycemic subjects and patients with prediabetes. **a** Gating strategy for the analysis of senescent T cells. **b**, **c** Representative flow cytometry plots are presented for CD57 and CD28 expression by CD4^+^ or CD8^+^ T cells in normoglycemic subjects (*n* = 40) and patients with prediabetes (*n* = 40). Statistical analysis of the population of CD28^−^ and CD57^+^ T cells in CD4^+^ and CD8^+^ T cells in the two groups. Data are expressed as mean ± SEM. Flow cytometry plots are representative of at least three independent experiments. **P < 0.01 compared with the corresponding controls

Next, we investigated the immunophenotype of T cells in PBMCs from normal controls and patients with prediabetes. PBMCs were first gated for single cells (forward scatter-area vs. forward scatter-height) and lymphocytes (forward scatter-area vs. side scatter-area). The lymphocyte population was then further analyzed for uptake of a fixable viability dye to determine the proportion of live cells, and stained for CD3. The surface expression of CD4 and CD8 was then determined in this gated population (Fig. 2a). To compare the immunosenescence of T cells in PBMCs from normal controls and patients with prediabetes, we evaluated the frequency of CD57^+^ and/or CD28^−^ T cells among the CD4^+^ and CD8^+^ T cells in the PBMCs from the study population. Among CD4^+^ T cells, the CD28^−^CD57^+^ senescent population tended to be larger in patients with prediabetes, but this difference did not reach statistical significance (Fig. 2b). The population of CD28^−^CD57^+^CD8^+^ T cells was also significantly larger in patients with prediabetes (Fig. 2c). We also analyzed the population of CD28^−^CD57^+^ senescent CD4^+^ and CD8^+^ T cells in the patients with prediabetes according to diagnostic criteria for prediabetes. There was no significant difference in the population of CD28^−^CD57^+^CD4^+^ T cells among groups of subjects with impaired fasting glucose (IFG), impaired glucose tolerance (IGT), and IFG plus IGT (Supplementary Fig. 3a). However, the population of CD28^−^CD57^+^CD8^+^ T cells was significantly larger in patients with IFG plus IGT than in patients with IFG alone (Supplementary Fig. 3b). Collectively, these data demonstrate that there are more immunosenescent CD8^+^ T cells in patients with prediabetes than in normoglycemic subjects.

### Proinflammatory cytokines and cytotoxic molecules are highly expressed in senescent T cells from patients with prediabetes

**Fig. 3:**
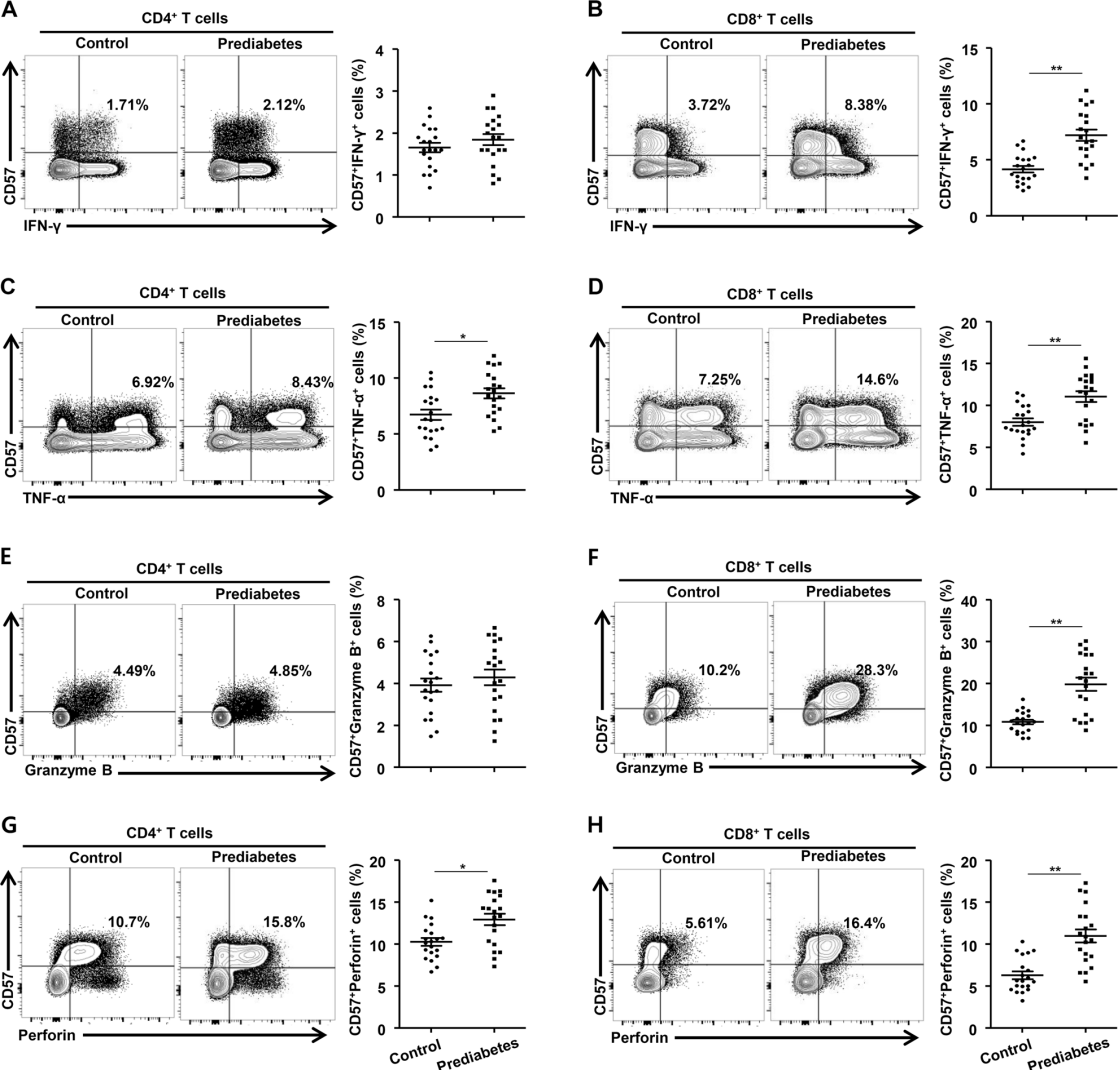
Functional characteristics of senescent T cells from patients with prediabetes and normoglycemic subjects. Intracellular immunostaining for proinflammatory cytokines and cytotoxic granule proteins was performed in CD4^+^ and CD8^+^ T cells in prediabetic and normoglycemic subjects. **a**–**d** The number of IFN-γ- and TNF-α-secreting cells in the population of CD57^+^CD4^+^ or CD8^+^ T cells was compared between the two groups (*n* = 20). **e**–**h** The numbers of granzyme B^+^ or perforin^+^ cells in the population of CD57^+^ CD4^+^ or CD8^+^ cells were evaluated by flow cytometry (*n* = 20). Data are expressed as mean ± SEM. *P < 0.05, **P < 0.01 compared with the corresponding controls. Flow cytometry plots are representative of at least three independent experiments

Senescent T cells have the ability to secret a large quantity of proinflammatory cytokines and cytotoxic molecules^22^. Therefore, we investigated the functional characteristics of senescent CD4^+^ and CD8^+^ T cells from normal controls and patients with prediabetes. IFN-γ, TNF-α, and IL-17A production was quantified in the senescent CD4^+^ and CD8^+^ T cells. In consistent with previous reports^23,24^, IFN-γ and TNF-α production in total CD4^+^ and CD8^+^ T cells were increased in patients with prediabetes (Fig. 3a–d). As shown in Supplementary Fig. 4a–d, the population of IFN-γ and TNF-α producing total CD4+ and CD8+ T cells are significantly increased in the PBMCs from the patients with prediabetes compared with normal subjects. Interestingly, we also detected significant increases in the production of IFN-γ and TNF-α in the senescent CD4^+^ and CD8^+^ T cells as well as in the CD57^−^ T cells (Fig. 3a–d). However, IL-17A production by senescent T cells was not detected in the PBMCs (Supplementary Fig. 5a, b). There was also no significant difference in IL-17A production between CD4^+^ and CD8^+^ T cells (Supplementary Fig. 5a, b). We also evaluated the expression of cytotoxic molecules in senescent CD4^+^ and CD8^+^ T cells from the study subjects. The population of granzyme B-producing senescent CD8^+^ cells was significantly larger in patients with prediabetes, but there was no significant difference in the expression of granzyme B in senescent CD4^+^ T cells between the two groups (Fig. 3e, f). The production of perforin was also greater in the senescent CD4^+^ and CD8^+^ cells from the patients with prediabetes (Fig. 3g, h). These data suggest that patients with prediabetes have a larger population of proinflammatory and cytotoxic senescent CD8^+^ T cells.

### Patients with prediabetes have higher concentrations of ROS and glycolytic profile in their senescent CD8^+^ T cells

**Fig. 4:**
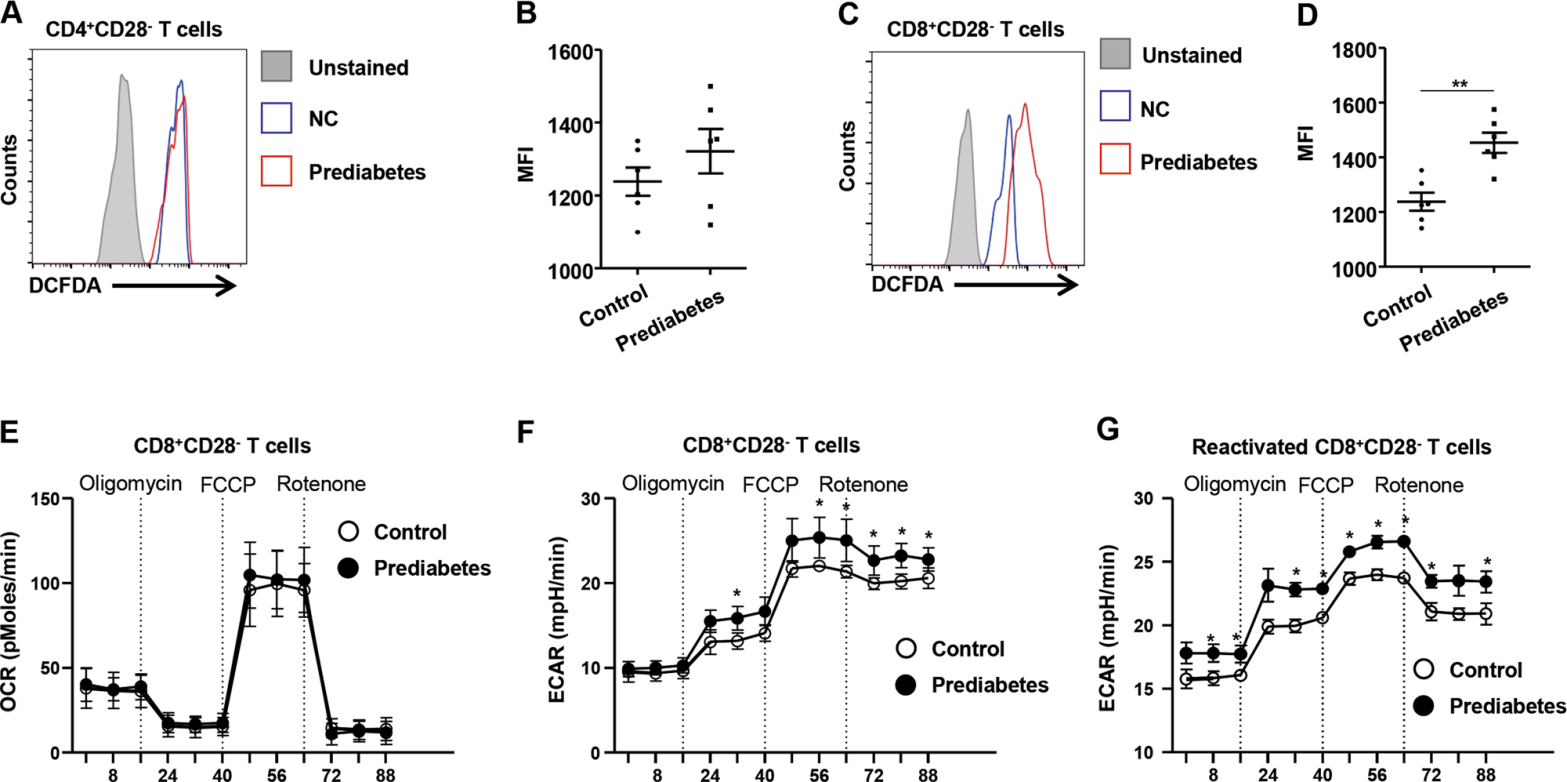
Metabolic reprogramming of senescent CD8^+^ T cells from patients with prediabetes. **a**, **b** The production of ROS was monitored by DCF fluorescence and quantified as the MFI in CD4^+^CD28^−^ T cells from normoglycemic subjects (*n* = 6) and patients with prediabetes (*n* = 6). **c**, **d** The production of ROS was monitored by DCF fluorescence and quantified as the MFI in CD8^+^CD28^−^ T cells from normoglycemic subjects (*n* = 6) and patients with prediabetes (*n* = 6). **e**, **f** Oxygen consumption rate and extracellular acidification rate (ECAR), measured in CD8^+^CD28^−^ T cells from normoglycemic subjects (*n* = 6) and patients with prediabetes (*n* = 6). **g** Extracellular acidification rate (ECAR), measured in activated CD8^+^CD28^−^ T cells from normoglycemic subjects (*n* = 6) and patients with prediabetes (*n* = 6). Data are expressed as mean ± SEM. *P < 0.05 compared with the corresponding controls

ROS are implicated in hyperglycemia-induced inflammation^25^. To investigate the relationship between hyperglycemia and ROS production in T cells, we measured ROS levels in the CD4^+^CD28^−^ T cells as well as the CD8^+^CD28^−^ T cells from normal subjects and patients with prediabetes. CD4^+^CD28^−^ T cells contained similar concentrations of ROS, regardless of serum glucose status (Fig. 4a, b), whereas CD8^+^CD28^−^ T cells from the patients with prediabetes had a higher capacity to produce ROS than those from normal subjects (Fig. 4c, d). Metabolic reprogramming orchestrates the functions of T cells^26^. Therefore, we measured the oxygen consumption rate (OCR) and extracellular acidification rate (ECAR) in CD8^+^CD28^−^ T cells from normal subjects and the patients with prediabetes, and found no marked difference in the OCR of CD8^+^CD28^−^ T cells between these groups (Fig. 4e). However, resting CD8^+^CD28^−^ T cells showed a typical glycolytic profile, characterized by high elevated ECAR, consistent with abnormal glucose homeostasis (Fig. 4f). We also found that activated CD8^+^CD28^−^ T cells using anti-CD3 antibodies showed a higher ECAR in the patients with prediabetes than in normal controls (Fig. 4g), suggesting that metabolically higher glycolytic potential of CD8^+^CD28^−^ T cells is visible in the patients with abnormal glucose homeostasis. These findings indicate that the larger quantity of ROS produced by metabolically reprogrammed CD8^+^CD28^−^ T cells is associated with impaired glucose homeostasis.

### T-cell senescence in prediabetes contributes to ATF5-mediated GDF15 expression in the liver

**Fig. 5:**
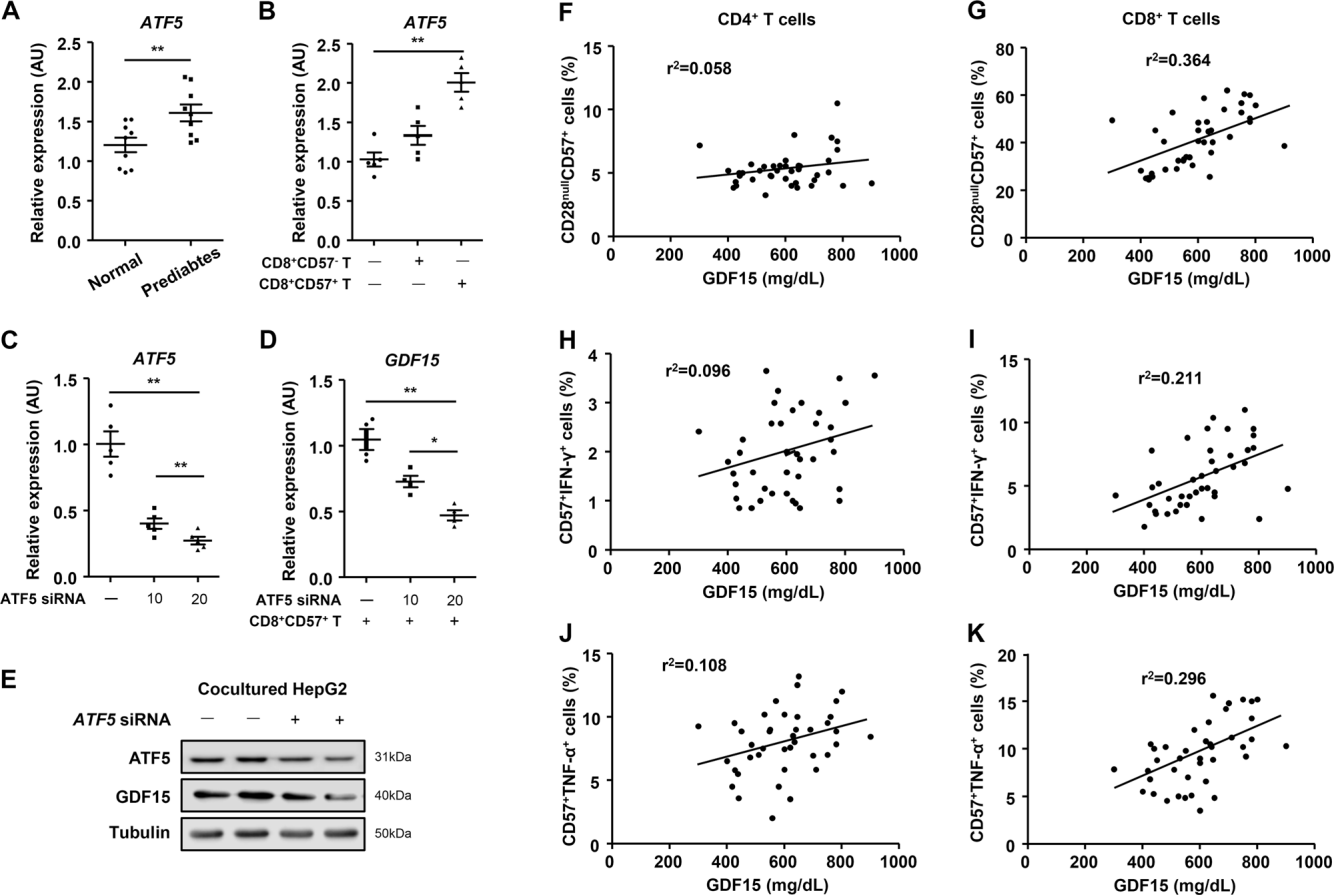
T-cell senescence is associated with ATF5-mediated GDF15 expression in the liver. **a** Liver tissues from controls (*n* = 9) and patients with prediabetes (*n* = 9) were subjected to real-time PCR. **b** Real-time PCR analysis of *ATF5* in HepG2 cells co-cultured with CD8^+^CD57^−^ T cells or CD8^+^CD57^+^ T cells. **c**, **d** Real-time PCR analysis of HepG2 cells co-cultured with CD8^+^CD57^+^ T cells after the addition of *ATF5* siRNA. **d** Western blot analysis of HepG2 cells co-cultured with CD8^+^CD57^+^ T cells after treatment with or without *ATF5* siRNA. **f**, **g** Correlation analysis of serum GDF15 level and number of senescent CD4^+^ and CD8^+^ T cells (*P* = 0.14, *P* = 0.05). **h**, **i** Correlation analysis of serum GDF15 level and number of IFN-γ secreting senescent CD4^+^ and CD8^+^ T cells (*P* = 0.04, *P* < 0.01). **j**, **k** Correlation analysis of serum GDF15 level and number of TNF-α-secreting senescent CD4^+^ and CD8^+^ T cells (*P* < 0.01, *P* < 0.01). Data are expressed as mean ± SEM. *P < 0.05, **P < 0.01 compared with the corresponding controls

Functional activation of senescent CD8^+^ T cells might be associated with the systemic inflammatory response in the body. Growth differentiation factor 15 (GDF15), a marker of oxidative stress and the inflammatory process, is a stress-responsive hepatokine that is increased in obesity, glucose intolerance, and cardiovascular diseases^8^. Activating transcription factor 5 (ATF5) is involved in liver-specific inflammatory response and translational regulation in response to environmental stresses^22,27^. We found that hepatic *ATF5* expression was higher in patients with prediabetes than in controls (Fig. 5a, and Supplementary Table. 1). Next, to investigate the effect of senescent CD8^+^ T cells on hepatic *ATF5* expression, we co-cultured CD28^+^CD57^+^ or CD28^−^CD57^+^ T cells with HepG2 cells using Transwell inserts. Interestingly, senescent CD8^+^ T cells induced *ATF5* expression in HepG2 cells (Fig. 5b). Moreover, knockdown of *ATF5* reduced the expression of GDF15 in HepG2 cells co-cultured with senescent CD8^+^ T cells (Fig. 5c-e). In addition, we analyzed the correlation between serum GDF15 level and senescent CD4^+^ or CD8^+^ T-cell numbers in the patients with prediabetes (Fig. 5f, g). Although we observed a weak correlation between the size of the population or the activation of senescent CD4^+^ T cells and the GDF15 level (Fig. 5f), the number of senescent CD8^+^ T cells was strongly correlated with serum GDF15 (Fig. 5g). Moreover, the production of IFN-γ and TNF-α by the senescent CD8^+^ T cells exhibited a significant positive correlation with serum GDF15 (Fig. 5h–k). These data indicate that systemic inflammatory process are associated with the increased population and functional activation of senescent CD8^+^ T cells in patients with prediabetes.

### Effect of senescent CD8^+^ T cells on hepatic gluconeogenesis

**Fig. 6:**
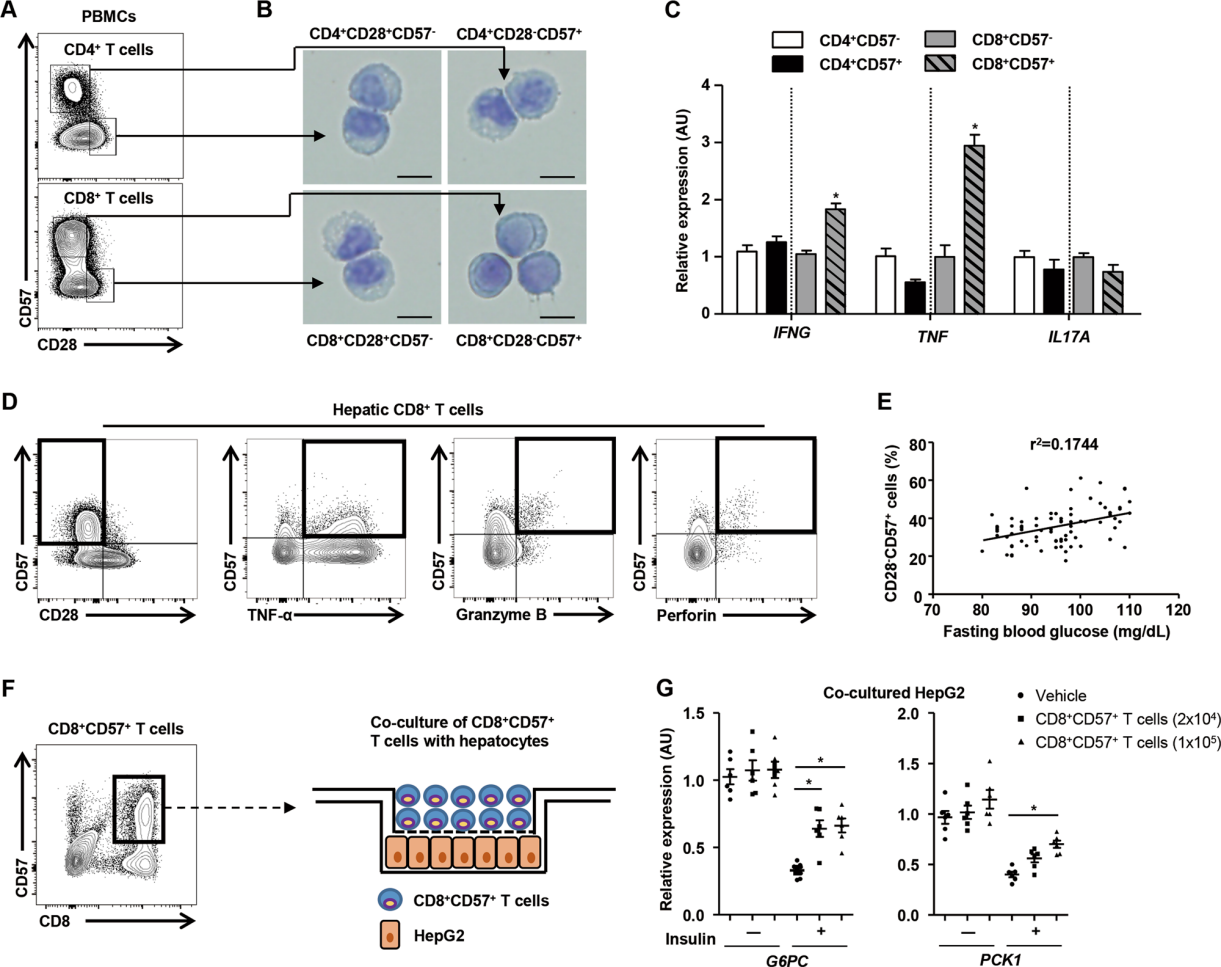
CD8^+^CD57^+^ T cells express larger quantities of proinflammatory cytokines and contribute to hepatic gluconeogenesis. **a** FACS analysis of human CD8^+^ T cells immunostained with anti-CD28 and anti-CD57 antibodies. **b** Subsets including CD28^−^CD57^+^- and CD28^+^CD57^−^-expressing human CD4^+^ and CD8^+^ T cells were visualized by Giemsa staining. Scale bar: 10 μm. **c** Four groups of the subsets were subjected to real-time PCR. **d** Population size and functional analysis of CD28^−^CD57^+^CD8^+^ T cells in human liver tissue. **e** Correlation between CD8^+^CD28^−^CD57^+^ T cells and fasting blood glucose in human subjects (*n* = 80; *P* < 0.01). Correlations were determined by Spearman correlation to assess the relationship (*P* < 0.01). **f** Schematic graphic of the co-culture of HepG2 with CD8^+^CD57^+^ T cells. **g** Real-time PCR analysis of *G6PC* and *PCK1* in HepG2 cells co-cultured with or without CD8^+^CD57^+^ T cells (2×10^4^ or 1×10^5^ cells) and insulin (100 nm). Results are representative of three independent experiments, and data are expressed as the mean ± SEM. *P < 0.05 compared with the corresponding controls. Flow cytometry plots are representative of at least three independent experiments

To characterize the morphology of senescent T cells, subsets of human CD4^+^ and CD8^+^ T cells, including CD28^−^CD57^+^ and CD28^+^CD57^−^, were sorted (Fig.6a) and then stained with Giemsa. The nuclei of all the cells were usually round but could also be oval or slightly indented, with light blue cytoplasm (Fig. 6b). There was no difference in size or granularity among the four subsets (Fig. 6b). However, CD8^+^CD28^−^CD57^+^ T cells showed higher expression of proinflammatory cytokines than other subsets of T cells in resting condition (Fig. 6c).

Hepatic immune cells may influence regional hepatocytes, thereby modulating hepatic glucose metabolism. Therefore, we sought to demonstrate the existence of and to functionally analyze senescent CD8^+^ T cells in the liver. We isolated and analyzed hepatic immune cells from human subjects using fluorescence-activated cell sorting (FACS), thereby confirming the presence of senescent CD8^+^ T cells in the liver (Fig. 6d). Like peripheral senescent CD8^+^ T cells, hepatic senescent CD8^+^ T cells also produced proinflammatory cytokine and cytotoxic molecules (Fig. 6d). The population of CD28^−^CD57^+^ senescent CD8^+^ T cells in liver was also significantly larger in patients with type 2 diabetes (Supplementary Fig. 6). Moreover, the number of senescent CD8^+^ T cells exhibited a positive correlation with fasting blood glucose (Fig. 6e). Next, to investigate the effect of hepatic senescent CD8^+^ T cells on hepatic gluconeogenesis, we co-cultured senescent CD8^+^ T cells with HepG2 cells using Transwell inserts (Fig. 6f). Real-time PCR analysis showed that treatment with insulin significantly reduced the expression of *G6PC* and *PCK1* in HepG2 cells (Fig. 6g). However, the suppression of gluconeogenic gene transcription by insulin was partially inhibited by co-culture with senescent CD8^+^ T cells (Fig. 6g). These data suggest that hepatic insulin sensitivity can be impaired by senescent CD8^+^ T cells.

### Senescent T cells are enriched in the liver of aged mice

Aging increases the prevalence of type 2 diabetes and systemic inflammation^28^, as well as the number of senescent T cells, in humans. Although the aging process is directly associated with the oligoclonal accumulation of CD8^+^CD28^−^ T cells in humans^29^, the age-related loss of CD28 from T cells is not usually observed in mice^30^. However, it has been shown that the expression of CD153 (also known as a TNF superfamily protein) and programmed cell death 1 in T cells increases with age in this species^31,32^. Therefore, we identified and characterized the senescent T cells in the liver of mice by FACS analysis using anti-CD153 and -CD279 antibodies. To determine the effect of aging on the size of the population of hepatic senescent T cells, we compared young (2-month-old) and old (16-month-old) mice. The old mice showed higher levels of fasting blood glucose and fasting insulin than the young mice (Supplementary Fig. 7a, b). We also found that primary hepatocytes isolated from 16-month-old mice showed an impairment in suppression of insulin-induced gluconeogenic gene transcription (Supplementary Fig. 7c). Interestingly, livers of aged mice have much higher numbers of CD4^+^CD44^+^CD153^+^ and CD8^+^CD44^+^CD153^+^ T cells (Supplementary Fig. 7d, e). Moreover, the numbers of CD4^+^CD44^+^CD279^+^ and CD8^+^CD44^+^CD279^+^ T cells in the liver were greater in old mice than young mice (Supplementary Figure 7f, g). The numbers of CD4^+^CD44^+^CD153^+^CD279^+^ and CD8^+^CD44^+^CD153^+^CD279^+^ T cells were also higher in the liver of old mice (Supplementary Fig. 7h, i). To evaluate the functional characteristics of the hepatic senescent T cells, we assessed TNF-α production by CD4^+^CD44^+^CD153^+^ and CD4^+^CD44^+^CD279^+^ T cells in the liver of young and old mice. TNF-α expression in the CD4^+^CD44^+^CD153^+^ and CD4^+^CD44^+^CD279^+^ T cells was significantly higher in the liver of old than young mice (Supplementary Fig. 7j–m). In old mice, the expression of TNF-α was also high in CD8^+^CD44^+^CD153^+^ and CD8^+^CD44^+^CD279^+^ T cells from the liver (Supplementary Fig. 7j–m). These results indicate that CD153^+^CD279^+^ T cells with features of inflammation are present in larger numbers in the livers of aged mice with glucose intolerance.

### Feeding a HFD induces T-cell senescence in mouse liver

**Fig. 7:**
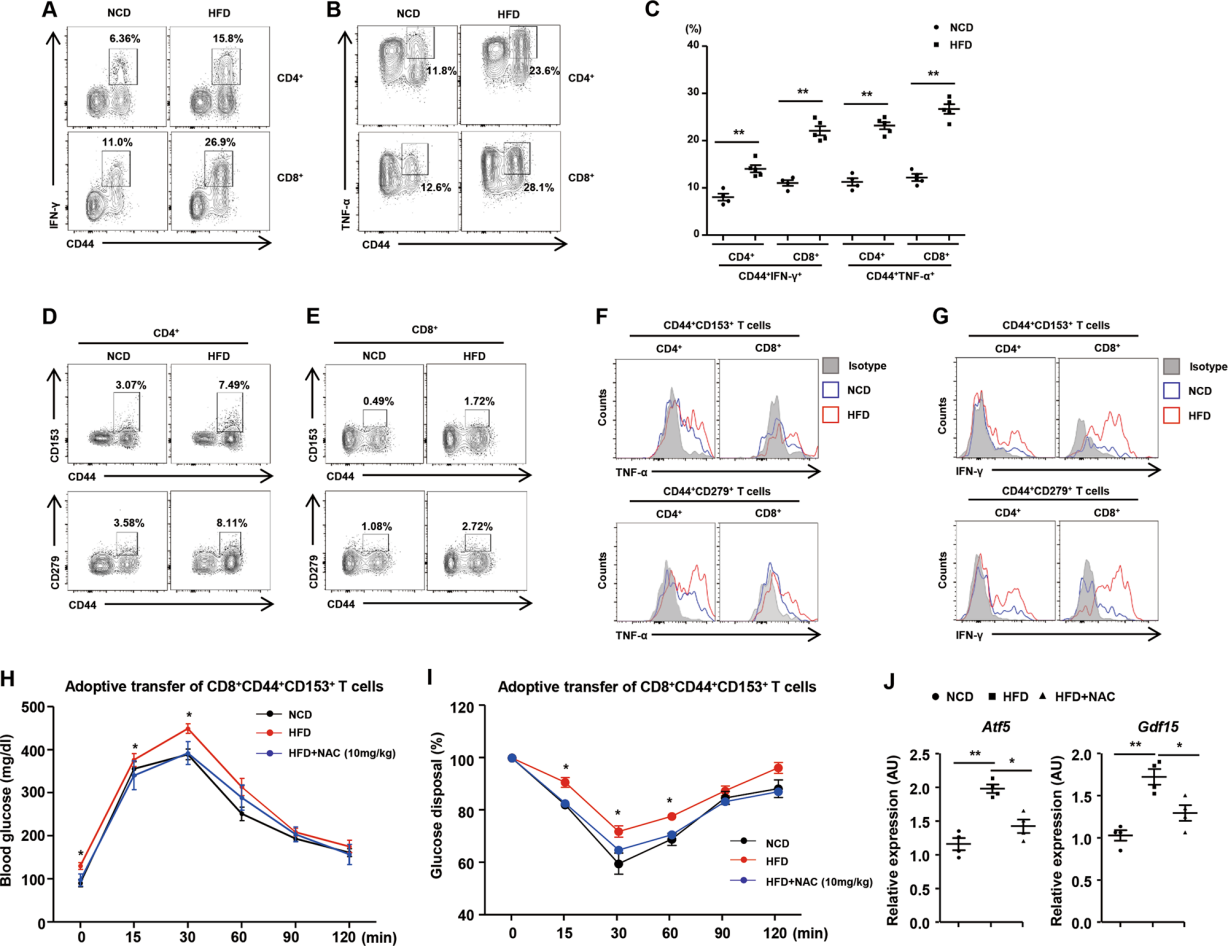
CD44^+^CD153^+^ and CD44^+^CD279^+^ subsets of CD4^+^ and CD8^+^ T cells occur in greater numbers in the liver of HFD-fed mice. **a**–**c** Populations of liver resident CD44^+^IFN-γ^+^ or CD44^+^ TNF-α^+^ in CD4^+^ and CD8^+^ T cells from mice fed a NCD (*n* = 4) or a HFD (*n* = 5). **d**, **e** FACS analysis of hepatic CD4^+^CD44^+^CD153^+^, CD8^+^CD44^+^CD153^+^, CD4^+^CD44^+^CD279^+^, and CD8^+^CD44^+^CD279^+^ T cells isolated from mice fed a NCD or a HFD. **f**, **g** TNF-α or IFN-γ expression of hepatic CD4^+^CD44^+^CD153^+^, CD8^+^CD44^+^CD153^+^, CD4^+^CD44^+^CD279^+^, and CD8^+^CD44^+^CD279^+^ T cells from mice fed a NCD or a HFD. **h**, **i** Effects of adoptive transfer of CD8^+^CD44^+^CD153^+^ T cells from mice fed a HFD (*n* = 4) with or without *N*-acetylcysteine (10mg/kg) or a NCD (*n* = 4) as determined by glucose tolerance and insulin tolerance test. Data are expressed as mean ± SEM. *P < 0.05, **P < 0.01 compared with the corresponding controls. Flow cytometry plots are representative of at least three independent experiments

Diet-induced obesity is associated with an altered hepatic immune microenvironment, which leads to metabolic deterioration in the liver^33^. Thus, to explore the effect of a high fat diet (HFD) on T-cell senescence in the liver, male C57BL/6J mice were fed either a normal chow diet (NCD) or a HFD for 8 weeks. We identified and further characterized senescent T cells in the liver of mice fed a HFD or a NCD. The mice fed a HFD showed a remarkable increase in the surface expression of CD44 and lower expression of CD62L in CD4^+^ and CD8^+^ T cells in their livers (Supplementary Fig. 8a, b). In addition, the populations of CD44^+^IFN-γ^+^ and CD44^+^TNF-α^+^ subsets were increased in CD4^+^ and CD8^+^ T cells from the livers of mice fed a HFD (Fig. 7a–c). The mice fed a HFD exhibited an increase in the population of infiltrating monocytes (CD11b^+^Ly6C^high^LyG^low^) in the livers (Supplementary Fig. 8c). We also found that the populations of CD44^+^CD153^+^ in CD4^+^ and CD8^+^ T cells were significantly larger in the livers of mice fed a HFD (Fig. 7d, e and Supplementary Fig. 9a). Moreover, the surface expression of CD279 in CD4^+^CD44^+^ and CD8^+^CD44 T cells was markedly higher in the livers of mice fed a HFD (Fig. 7d, e and Supplementary Fig. 9a). The expression of TNF-α and IFN-γ was also higher in CD44^+^CD153^+^ and CD44^+^CD279^+^ of CD4^+^ as well as CD8^+^ T cells from the livers of mice fed a HFD than in those from the livers of mice fed a NCD (Fig. 7f, g). Next, we investigated the migration of senescent CD8^+^ T cells in metabolic organs including liver and adipose tissue. Senescent CD8^+^ T cells were isolated from male CD45.1^+^ mice and transferred to CD45.2^+^ mice. We found that ~ 1% of adoptively transplanted senescent CD8^+^ T cells accumulated in the liver (Supplementary Fig. 9b, c), but we could not detect transplanted cells in the epididymal adipose tissues of the recipient mice (Supplementary Fig. 9b, c). To further confirm the immunometabolic role of senescent CD8^+^ T cells in the liver, we adoptively transferred CD8^+^CD44^+^CD153^+^ T cells isolated from the spleens of mice fed a HFD or a NCD into young (2-month-old) mice via tail vein. Adoptive transfer of CD8^+^CD44^+^CD153^+^ T cells reduced glucose disposal rates in young mice (Supplementary Fig. 9d). Moreover, the recipients of CD8^+^CD44^+^CD153^+^ T cells from mice fed a HFD exhibited significant aggravation of glucose tolerance compared to control mice transferred from mice fed a NCD (Fig. 7h). Adoptive transfer of CD8^+^CD44^+^CD153^+^ T cells also led to a significant deterioration in insulin sensitivity in mice (Supplementary Fig. 9e). CD8^+^CD44^+^CD153^+^ T cells from the mice fed a HFD exacerbated insulin resistance than those from the mice fed a NCD (Fig. 7i). Reducing ROS with *N*-acetylcysteine improves abnormal glucose homeostasis caused by injection of CD8^+^CD44^+^CD153^+^ T cells from the mice fed a HFD (Fig. 7h, i). Hepatic expression of *Atf5* and *Gdf15* in mice injected with CD8^+^CD44^+^CD153^+^ T cells from the mice fed a HFD was remarkably reduced by treatment with *N*-acetylcysteine (Fig. 7j). Furthermore, we co-cultured the murine hepatocytes with hepatic senescent CD8^+^ T cells using Transwell inserts in in vitro (Supplementary Fig. 10a). Treatment with *N*-acetylcysteine reduced the transcription of *Atf5* and *Gdf15* in primary hepatocytes co-cultured with hepatic senescent CD8^+^ T cells (Supplementary Fig. 10b). We also treated primary hepatocytes with conditioned media of hepatic senescent CD8^+^ T cells treated with *N*-acetylcysteine or vehicle. Conditioned media from hepatic senescent CD8^+^ T cells treated with *N*-acetylcysteine decreased the transcription of *Atf5* and *Gdf15* in hepatocytes (Supplementary Fig. 10c). This finding supports that ROS production by senescent CD8^+^ T cells contributes to mitochondrial stress response in the hepatocytes. These findings implicate distinct effects of murine T-cell senescence on obesity and abnormal glucose homeostasis.

## Discussion

Chronic inflammation is closely associated with a higher risk of cardiovascular disease, and seems to contribute to insulin resistance in patients with prediabetes^21^. Moreover, in a large representative population sample, immune and inflammatory responses were strongly stimulated in subjects with prediabetes^34^. We also showed in the present study that molecules involved in T-cell activation were upregulated in PBMCs from patients with prediabetes. In previous studies, T cells have been implicated in the pathogenesis of insulin resistance in rodents and humans^24,35–38^, and have been shown to contribute to the progression of tissue inflammation through the release of proinflammatory cytokines, such as IFN-γ and TNF-α^39^.

Although T cells proliferate by clonal expansion and differentiate into memory or effector T cells throughout the lifetime of an organism, impairment in memory formation by antigen-specific T cells leads to senescence or exhaustion of T cells, according to the size of the antigenic load or the degree of stimulation^40^. Although the role of senescent CD28^−^ T cells, a population of terminally differentiated memory T cells, is not fully understood, they have been shown to be more numerous in the presence of cardiovascular diseases and immune disorders^10,41^. In addition, treatment with a statin significantly reduces the number of peripheral CD4^+^CD28^−^ T cells and IFN-γ production by CD4^+^CD28^−^ T cells in patients with acute coronary syndrome^42,43^. In the present study, the population of peripheral CD28^−^CD57^+^CD8^+^ T cells was also larger in patients with prediabetes than in normal controls. The expression of proinflammatory cytokines and cytotoxic enzymes was also significantly higher in CD28^−^CD57^+^CD8^+^ T cells from patients with prediabetes. Although there is no difference in the population of senescent CD4^+^ T cells between patients with prediabetes and control subjects, the population of senescent CD4^+^ T cells producing TNF-α and Perforin is significantly increased in patients with prediabetes compared with control subjects. This discrepancy between population and function of senescent CD4^+^ T cells might originate from disease severity. It is also possible that changes in the population of senescent CD4^+^ T cells may follow the functional changes of senescent CD4^+^ T cells in the development of type 2 diabetes in humans. Patients with overt type 2 diabetes might show higher population of senescent CD4^+^ and CD8^+^ T cells in PBMCs compared with normal controls. However, further studies are needed to validate whether hyperglycemia progression increases the population of senescent CD4^+^ T cells in PBMCs.

During an immune response, the metabolic shift from oxidative phosphorylation to glycolysis supports the activation and proliferation of CD4^+^ and CD8^+^ T cells. A recent study also revealed that human CD8^+^CD28^−^ T cells have an increased capacity to use glycolysis, associated with the loss of SIRT1^44^. Moreover, intracellular ROS are necessary for driving and maintaining T-cell activation-induced metabolic reprogramming^45^. Oxidative stress is also induced in hyperglycemia, and the overproduction of ROS contributes to the pathogenesis of diabetic complications^46^. Therefore, metabolic switching by a senescent population of terminally differentiated T cells can be implicated in the production of ROS and proinflammatory cytokines, which leads to a systemic inflammatory response and metabolic deterioration. Consistent with previous reports, we found that CD8^+^CD28^−^ T cells had a higher capacity to produce ROS and exhibited glycolytic profile in patients with abnormal glucose homeostasis compared with normal subjects. In addition, we demonstrated that these CD8^+^CD28^−^ T cells reside within the liver and are more numerous in the liver of aged and HFD-fed mice. These suggest that T-cell senescence-mediated reprogramming of metabolic pathways may be associated with development of diabetes and its complications, as well as other aging-related diseases.

- In conclusion, we identified a link between the numbers and secretion of effector molecules by senescent T cells, and blood glucose status, in humans and mice. We also present evidence that senescent CD8^+^ T cells may contribute to inflammatory response during the development of diabetes. Finally, we demonstrated that the presence of hepatic senescent T cells is associated with abnormal glucose homeostasis in aged and diet-induced obese mice. Therefore, this study defines a new clinically relevant concept of a T-cell senescence-mediated inflammatory response in the pathophysiology of diabetes.

## Materials and methods

### Study population

We recruited 80 participants who did not have diabetes mellitus in Chungnam National University Hospital between October 2015 and April 2017. Patients with any of the following conditions were excluded from the study: previous coronary heart disease, history of any arrhythmia, malignant hypertension, severe pulmonary disease, acute or chronic kidney disease (estimated glomerular filtration rate < 30 mL/min/1.73 m^2^), anemia (hemoglobin < 12 g/dL), history of any malignant or inflammatory disease, current liver disease, or high plasma aspartate transaminase or alanine transaminase (> 80 IU/L). The participants were divided into two groups: normoglycemic (*n* = 40) and prediabetic (*n* = 40). Age- and gender-matched, healthy control subjects and patients with prediabetes were randomly selected and evaluated by history taking, physical examination, laboratory testing, and flow cytometry analysis. Prediabetes was defined by the presence of the following: (i) IFG (blood glucose: 100–125 mg/dL); (ii) IGT (blood glucose: 140–199 mL/dL after a 2 h oral glucose tolerance test); and (iii) glycated hemoglobin of 5.7–6.5%. Liver biopsies from the patients did not contain tumor cells and showed no signs of viral hepatitis.

This study was reviewed and approved by the Institutional Review Board of Chungnam National University Hospital (CNUH 2015-09-042), according to the standards of the Declaration of Helsinki. Written and oral informed consent, documented by the Department of Internal Medicine of Chungnam National University Hospital in South Korea, was obtained from all of the participants prior to their inclusion in the study.

### Mice

Male C57BL/6 wild-type mice were purchased from the Jackson Laboratory (Bar Harbor, ME, USA). All mice were maintained in a controlled environment (12 h light/12 h dark cycle; humidity: 50–60%; ambient temperature: 22±2 °C) in a specific pathogen-free animal facility at the Chungnam National University Hospital Preclinical Research Center. Mice were fed either a NCD or a HFD (Research Diets. Inc., New Brunswick, NJ, USA), starting when the mice were 6-week-old, and continuing for 8 weeks. Young (2-month-old) and old (16-month-old) mice were also used to investigate the effect of aging on the senescence of murine T cells in the liver. All animals received humane care according to the criteria outlined in the Guide for the Care and Use of Laboratory Animals, published by the National Institutes of Health, and all experimental procedures were approved by the Institutional Animal Care and Use Committee of the Chungnam National University School of Medicine.

### Preparation of PBMCs

Peripheral blood samples (8–10 mL each) were obtained from all participants, transferred aseptically into 50 mL polystyrene centrifuge tubes containing ethylenediaminetetraacetic acid (Sigma-Aldrich, Dorset, UK) as an anticoagulant, and gently mixed. PBMCs were isolated by centrifugation on a Ficoll-Paque density gradient (GE Healthcare Life Science, Buckinghamshire, UK) at room temperature. After centrifugation, the PBMC layer was collected and washed in Dulbecco’s phosphate-buffered saline . The isolated PBMCs were resuspended in 2 mL Rosewell Park Memorial Institute (RPMI)-1640 medium (Welgene, Daegu, South Korea), and trypan blue dye exclusion testing was used to determine the number of viable cells in the suspension.

### FACS analysis of human PBMCs

PBMCs were incubated with directly fluorochrome-conjugated monoclonal antibodies for 40 min at 4 °C. The antibodies used in this study were anti-CD3-PerCP-Cy5.5, anti-CD3-PE-Cy7, anti-CD4-AF700, anti-CD8-PE, anti-CD8-APC, anti-CD28-APC, anti-CD57-FITC, fixable viability dye-APC-Cy7, anti-IFN)-γ-PE-Cy7, anti-TNF-α-APC, anti-IL-17A-APC, anti-perforin-PerCP-Cy5.5, anti-granzyme B-PE, and anti-FVD-APC-Cy7 (all supplied by eBioscience, San Diego, CA, USA). PBMCs were stimulated with phorbol-myristate acetate/ionomycin/brefeldin A/monensin for 5 h. The cells were fixed and permeabilized using a Fixation/Permeabilization Buffer kit (eBioscience, San Diego, CA, USA). The permeabilized cells were washed and resuspended in 1% formaldehyde and further stained for intracellular cytokines and cytotoxic molecules with anti-IFN-γ-PE-Cy7, anti-TNF-α-APC, anti-IL-17A-APC, anti-perforin-PerCP-Cy5.5, and anti-granzyme B-PE. Multicolor flow cytometry was performed using a BD FACSCanto II Flow Cytometer (BD Biosciences, San Jose, CA, USA), and the data were analyzed using FlowJo software (Tree Star, Ashland, OR, USA).

### Microarray analysis using bioinformatic tools

Total RNA was prepared from PBMCs obtained from patients who had been newly diagnosed with diabetes, and age- and sex-matched control subjects. RNA amplification and labeling were performed with a Low RNA Input Linear Amplification kit PLUS (Agilent Technologies, Santa Clara, CA, USA). Array hybridization and scanning were performed with a DNA microarray chip and scanner (Agilent Technologies). Data were analyzed using Feature Extraction and GeneSpring Software (Agilent Technologies). The Database for Annotation, Visualization, and Integrated Discovery (DAVID) of the NIH was approved to detect functional gene annotation clusters, based on gene expression profiling by gene annotation enrichment analysis. Moreover, differentially expressed genes were then subjected to hierarchical clustering and phenotype ontology using Network2Canvas (http://maayanlab.net/N2C/). Phenotype categories were visualized on the grid according to gene-list similarity, with enriched categories being indicated by circles. In addition, Gene Set Enrichment Analysis (http://www.broadinstitute.org/gsea) was performed on transcriptome data for PBMCs from normal subjects and patients with prediabetes. Microarray analysis was carried out with R package v3.2.5, available at http://www.r-project.org. A heatmap was produced by color-coding standardized log gene expression levels (mean, zero; standard deviation, one). Probesets are shown hierarchically clustered by similarity, based on Euclidean distance and the Ward aggregation algorithm.

### Measurement of ROS in PBMCs

The PBMCs (2 × 10^5^ cells) were washed with PBS and incubated with surface immune-fluorescence-conjugated antibodies for 30 min. The cells were incubated with the ROS detection reagent 6-chloromethyl-2′,7′-dichlorodihydrofluorescein diacetate, acetyl ester (CM-H2DCF-DA from Invitrogen, Carlsbad, CA, USA) at 37 °C for 15 min, while other cells incubated with PBS were used as negative controls. Oxidation of the CM-H2DCF-DA probe was analyzed by excitation at 492–495 nm and the monitoring of the fluorescent emission at 517–527 nm using a BD FACSCanto II Flow Cytometer (BD Biosciences, San Jose, CA, USA). A histogram of the relative fluorescence intensities was used to compare intracellular ROS generation in PBMC subsets.

### Measurement of OCR and ECAR

Mitochondrial OCR and ECAR were determined using a Seahorse XF-96 Extracellular Flux Analyzer (Seahorse Bioscience Inc., North Billerica, MA, USA) in 96-well plates. CD8^+^CD28^−^ T cells were sorted by FACS Aria II (BD Bioscience, San Jose, CA, USA) using surface immune-fluorescence-conjugated antibodies. Resting CD8^+^CD28^−^ T cells were seeded onto Seahorse XF-96 plates at a density of 50,000 cells per well. The cells were washed and incubated with RPMI-1640 medium lacking sodium bicarbonate at 37 °C in a non-CO_2_-containing incubator for 1 h. The medium and mitochondrial OXPHOS inhibitors were adjusted to pH 7.4 on the day of the OCR assay. Measurements of OCR and ECAR were obtained under basal conditions and after adding 1 µm oligomycin, 0.5 µm FCCP, and 0.5 µm rotenone. OCR and ECAR were automatically calculated and recorded by the sensor cartridge and Seahorse XF-96 software.

### Hepatocyte isolation

As described previously^47,48^, murine hepatocytes were isolated from mice by in situ collagenase perfusion, followed by differential centrifugation on a Percoll (GE Healthcare, Buckingham, UK) density gradient, after filtering the cell suspension using a 70 μm nylon mesh (BD Falcon, NJ, USA). Isolated hepatocytes were cultured in low glucose (1.0 g/L) Dulbecco’s modified Eagle’s medium supplemented with 10% fetal bovine serum (Invitrogen, Carlsbad, CA, USA) and 1% penicillin/streptomycin (Invitrogen, Carlsbad, CA, USA) at a density of 1×10^5^ cells/well on 12-well plates.

### Adoptive transfer of CD8^+^CD44^+^CD153^+^ T cells into recipient mice

CD8^+^CD44^+^CD153^+^ T cells were isolated from the spleen of mice fed a HFD or a NCD for 8 weeks. A total of 1 × 10^6^ cells diluted with RPMI medium were injected to young (2-month-old) mice through tail vein, two times per week for 2 weeks. The vehicle was also similarly injected into the control young (2-month-old) mice. *N*-acetylcysteine (Sigma-Aldrich, St. Louis, MO, USA) was administered intraperitoneally at a dose of 10 mg/kg dissolved in 0.5 mL of phosphate-buffered saline three times per week for 2 weeks.

### Statistical analysis

All continuous variables are reported as the mean ± standard deviation. Statistical significance was defined as *P* < 0.05 and obtained using two-tailed Student’s *t* test or two-way analysis of variance, as appropriate. Categorical variables were calculated as a percentage of the group total. To analyze the strength of the relationship between continuous variables, Pearson correlation coefficients were used. Calculation of the statistical significance was based on the assumption that values exhibit a Gaussian distribution. Statistical analysis was performed using Graph Pad PRISM 6 or R package v3.2.5.

## Supplementary information

Supplemental materials and methods

Supplemental data

Supplemental figure legends
