# Supplementary material for: T-cell senescence contributes to abnormal glucose homeostasis in humans and mice
Source: Cell Death Dis. 2019 Mar 13;10(3):249. doi: 10.1038/s41419-019-1494-4 (PMC6416326; doi:10.1038/s41419-019-1494-4)
Supplement: Supplementary file 1 — Supplemental materials and methods [file 41419_2019_1494_MOESM1_ESM.docx]

**Supplemental Information**

**T cell senescence contributes to abnormal glucose homeostasis in humans and mice**

- Hyon-Seung Yi^1,2^*^#^, So Yeon Kim^3,4#^, Jung Tae Kim^1,5^, Young-Sun Lee^6^, Ji Sun Moon^1^, Mingyo Kim^7^, Yea Eun Kang^1,2^, Kyong Hye Joung^1,2^, Ju Hee Lee^1,2^, Hyun Jin Kim^1,2^, Kwangsik Chun^8^, Minho Shong^1,2^, Bon Jeong Ku^1,2^*
- ^1^Research Center for Endocrine and Metabolic Diseases, Chungnam National University Hospital, Chungnam National University School of Medicine, Daejeon 35015, Republic of Korea. ^2^Department of Internal Medicine, Chungnam National University School of Medicine, Daejeon 35015, Republic of Korea. ^3^Laboratory of Liver Research, Biomedical Science and Engineering Interdisciplinary Program, Korean Advanced Institute of Science and Technology, Daejeon, 34141, Republic of Korea. ^4^Division of Digestive and Liver Diseases, Department of Medicine Cedars-Sinai Medical Center, Los Angeles, CA 90048. ^5^Department of Medical Science, Chungnam National University School of Medicine, 266 Munhwaro, Daejeon 35015, Republic of Korea. ^6^Department of Internal Medicine, Korea University College of Medicine, Seoul, 08308, Republic of Korea. ^7^Division of Rheumatology, Department of Internal Medicine, Gyeongsang National University School of Medicine, 79, Gangnam-ro, Jinju, Gyeongnam 660-702, Republic of Korea. ^8^Department of Surgery, Chungnam National University School of Medicine, Daejeon 35015, Republic of Korea
- ^#^ These authors contributed equally to this work.

**Supplemental Materials and Methods**

**Human liver samples**

Liver tissues were from 10 subjects who underwent hepatectomy at Chungnam National University Hospital due to hepatocellular carcinoma or metastatic liver cancer. Clinical characteristics of all subjects are described in Supplementary Table 1. The non-tumor areas were isolated and used for FACS analysis. Written informed consent was obtained from all subjects. This protocol and study were approved by the Institutional Review Board of Chungnam National University Hospital (CNUH 2015-04-014). All experiments were performed in accordance with relevant guidelines and regulations.

**Preparation of liver MNCs and FACS analysis**

Livers were removed from the mice, minced into small pieces, and then put into gentleMACS C Tubes (Miltenyi Biotec, Auburn, CA, USA) with 5 mL DMEM digestion solution. Liver MNCs were isolated using a gentleMACS Dissociator (Miltenyi Biotec, Auburn, CA, USA), according to the instructions provided by the manufacturer. Isolated liver MNCs were counted and resuspended in DPBS containing 0.5% BSA and 0.05% sodium azide. The cells were pre-incubated with anti-mouse CD16/32 Fc blocker (BD Pharmingen, USA), prior to the addition of antibodies, to block non-specific reactions. The cells were stained with fluorescence-conjugated anti-CD45, anti-CD3e-FITC, anti-CD4-BV786, anti-CD8-APC-Cy7, anti-CD44-PerCP-Cy5.5, anti-CD153-PE, anti-CD279-PE-Cy7, and anti-TNF-α-APC antibodies (all supplied by eBioscience, San Diego, CA, USA). The immunostained cells were analyzed using a BD FACSCanto II Flow Cytometer (BD Biosciences, San Jose, CA, USA) and FlowJo software (Tree Star, Ashland, OR, USA).

**In vitro stimulation of human T cells and intracellular immunostaining**

For intracellular staining, cells were re-stimulated with phorbol-myristate acetate/ionomycin for 1 h. After 1 h of incubation in 96-well cell culture plates, brefeldin A and monensin (Protein Transport Inhibitor Cocktail; eBioscience, San Diego, CA, USA) were added, to prevent protein secretion. Following surface immunostaining with anti-CD3-PerCP-Cy5.5, anti-CD3-PE-Cy7, anti-CD4-AF700, anti-CD8-PE, anti-CD25-APC, and anti-CD57-FITC, the cells were fixed and permeabilized using the Fixation/Permeabilization Buffer kit and immunostained for intracellular cytokines with anti-IFN-γ-PE-Cy7, anti-TNF-α-APC, and anti-IL-17A-APC. Cytotoxic granule proteins were also immunostained using anti-perforin-PerCP-Cy5.5 and anti-granzyme B-PE antibodies (all supplied by eBioscience, San Diego, CA, USA).

**Isolation and Giemsa staining of senescent T cells**

To isolate senescent CD4^+^ and CD8^+^ T cells from human PBMCs, all resuspended cells were further sorted by FACS Aria II (BD Bioscience, San Jose, CA, USA) using anti-CD3-PerCP-Cy5.5, anti-CD3-PE-Cy7, anti-CD4-AF700, anti-CD8-PE, anti-CD25-APC, and anti-CD57-FITC antibodies (eBioscience, San Diego, CA, USA). The four groups of sorted cells including CD4^+^CD28^+^CD57^−^, CD4^+^CD28^−^CD57^+^, CD8^+^CD28^+^CD57^−^, and CD8^+^CD28^−^CD57^+^ T cells were stained with Giemsa solution for evaluation of cellular morphology.

**Biochemical data**

Peripheral blood was collected into heparin-coated tubes in the morning after an overnight fast. Blood glucose was measured, and the lipid profile (low-density lipoprotein cholesterol, high-density lipoprotein cholesterol, total cholesterol, and triglyceride) was evaluated using a blood chemistry analyzer (Hitachi 47; Hitachi, Tokyo, Japan). Plasma insulin was measured using an immunoradiometric assay kit (DIAsource INS-IRMA kit, DIAsource, Louvain-la-Neuve, Belgium). Glycosylated hemoglobin was quantified by high-performance liquid chromatography (BioRad, Hercules, CA, USA). Aspartate transaminase and alanine transaminase activities were measured using the International Federation of Clinical Chemistry Ultra Violet method without pyridoxal phosphate (TBA-2000FR; Toshiba, Tokyo, Japan). High-sensitivity C-reactive protein was measured by the photometric latex agglutination method (TBA-2000FR; Toshiba). Homeostatic model assessment-insulin resistance (HOMA-IR) was calculated as fasting serum insulin (µU/mL) × fasting plasma glucose (mmol/L) / 22.5.

**Correlation between serum levels of GDF15 and senescent T cells**

Hepatic *GDF15* expression and GDF15 levels in serum were measured, and senescent T cells and their INF-γ or TNF-α expression in PBMCs were also determined in the participants. The correlations between serum levels of GDF15, and the numbers of senescent CD4^+^ and CD8^+^ T cells, were analyzed using Graph Pad PRISM 6.

**Small Interfering Ribonucleic Acid (siRNA) Targeting *ATF5* and Transfection of HepG2 cells**

Human *ATF5* siRNA (ID: 108261) were purchased from Thermo Fisher Scientific. HepG2 cells were seeded into 6-well plates at a density of 3× 10^5^ cells/well, and then were transfected with *ATF5* siRNA or negative scramble siRNA using Lipofectamine™ RNAiMAX (Invitrogen, CA, USA) at various siRNA concentrations for 7 hours. The transfected cells were incubated in medium containing 10% fetal bovine serum, and then the cells were co-cultured with or without CD8^+^CD57^+^ T cells using Transwell insert for 48 hours.

**Western blot analysis**

HepG2 cells treated with *ATF5* siRNA or negative scramble siRNA were homogenized in RIPA buffer (30 mmol/L Tris, pH 7.5, 150 mmol/L sodium chloride, 1 mmol/L phenylmethylsulfonyl fluoride, 1 mmol/L sodium orthovanadate, 1% Nonidet P-40, 10% glycerol, phosphotase and protease inhibitors) for extraction of the protein. Western blot analysis of protein (30–50 µg) was performed according to standard procedures using the following commercially available antibodies: anti-ATF5 (Abcam, Cambridge, MA, USA; ab184923), anti-GDF15 (Santa Cruz, CA, USA; sc-66905), and anti-α-tubulin (Sigma-Aldrich, ST. Louis, MO, USA; T5168). Immunoreactive bands were visualized on nitrocellulose membranes using alkaline-phosphate-linked anti-rabbit antibody and the ECL detection system with Image Studio Digits (LI-COR Biosciences, Lincoln, NE, USA).

**Co-culture of CD8^+^CD28^−^ T cells with hepatocytes**

Splenic mononuclear cells (MNCs) were isolated by passing splenic tissue through a cell strainer with 70 µm nylon mesh. Red blood cells (RBCs) were lysed using RBC lysis buffer, and then CD8^+^ T cells were enriched using Magnisort negative selection magnetic beads, according to the manufacturer’s instructions (eBioscience, San Diego, CA, USA). To obtain CD8^+^CD28^−^ T cells, sorted CD8^+^ T cells were stained with biotinylated anti-CD28 antibodies, and then CD8^+^CD28^−^ T cells were purified using Magnisort negative selection magnetic beads (eBioscience, San Diego, CA, USA). Primary hepatocytes were seeded onto collagen-coated 12-well plates and cultured at a density of 1 × 10^5^ cells/well for 24 h. Freshly isolated CD8^+^CD28^−^ T cells (1 × 10^5^ cells/well) were co-cultured on a Transwell insert for 6 h.

**Intraperitoneal glucose tolerance test**

The recipient mice were fasted for 16 h prior to the glucose tolerance test. Next, 2 g of glucose per kilogram of body weight was injected into the intraperitoneal cavity and blood glucose levels were measured with a glucometer (Accu-CHEK Active, Roche Diagnostics, IN, USA) at 0, 15, 30, 60, 90, and 120 min.

**Intraperitoneal insulin tolerance test**

For insulin tolerance test, mice were fasted for 6 h prior to receiving an intraperitoneal injection of 0.75 U/kg insulin (Humalog, Eli Lilly, Indianapolis, IN, USA) and blood glucose levels were measured at 15, 30, 60, 90, and 120 min.

**Real-time PCR**

Real-time PCR analysis was performed using the primers listed in Supplementary Table 2. Total RNA was extracted from PBMCs using TRIzol Reagent (Invitrogen, Eugene, OR, USA), in accordance with the manufacturer’s instructions. cDNA was synthesized from the same quantity of RNA with M-MLV reverse transcriptase and oligo-dT primers (Invitrogen, Eugene, OR, USA), following the manufacturer’s protocol. Real-time PCR was performed using QuantiTect SYBR Green PCR Master Mix (Qiagen, Valencia, CA, USA) and analyzed on an ABI Prism 7000 Sequence Detection System (Applied Biosystems, Foster City, CA, USA). The comparative Ct method was used to quantify transcripts, expression of which was normalized to β-actin expression. The results were analyzed using the ΔΔCt method, and values are expressed as fold differences from control.

**Co-culturing hepatocyte with hepatic senescent T cells**

Mouse hepatocytes cells were cultured in the lower chambers of 12-well plates for 1 day. Isolated hepatic senescent CD8^+^ T cells were cultured in the upper chambers of 12-well plates with N-acetylcysteine (5 mM) or vehicle for 6 hours. Then, the hepatocytes and hepatic senescent CD8^+^ T cells were co-cultured in the Transwell inserts (Corning Inc., Corning, NY) for 6 hours. Moreover, the hepatocytes were treated with conditioned media of hepatic senescent CD8^+^ T cells treated with NAC or vehicle. The hepatocytes co-cultured or treated with conditioned media were subjected to real-time PCR analysis.

**Adoptive transfer of CD45.1^+^ senescent CD8^+^ T cells into recipient mice**

Senescent CD8^+^ T cells were isolated from male CD45.1^+^ mice and transferred to CD45.2^+^ mice. 1 x 10^6^ senescent CD8^+^ T cells were injected to age-matched male CD45.2^+^ mice through tail vein. After 12 hours, the mice were sacrificed, and then liver and adipose tissues were extracted for FACS analysis of tissue resident immune cells. Proportion of CD45.1^+^ senescent CD8^+^ T cells in the livers and adipose tissues of the recipients were displayed using FACS plots.
