# Supplementary material for: T-cell senescence contributes to abnormal glucose homeostasis in humans and mice
Source: Cell Death Dis. 2019 Mar 13;10(3):249. doi: 10.1038/s41419-019-1494-4 (PMC6416326; doi:10.1038/s41419-019-1494-4)
Supplement: Supplementary file 2 — Supplemental data [file 41419_2019_1494_MOESM2_ESM.pdf]

Supplementary Fig. 1

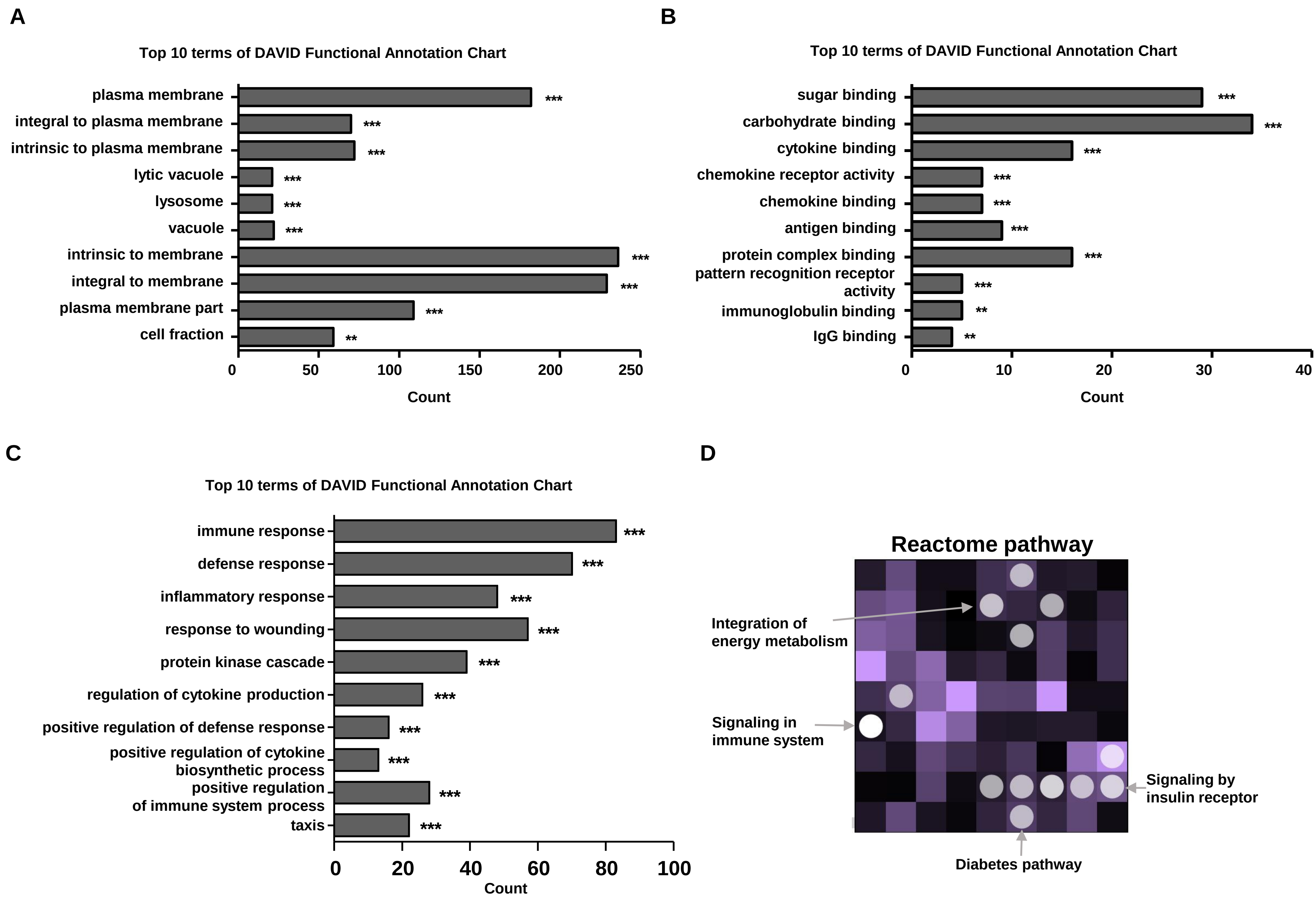

Supplementary Fig. 2

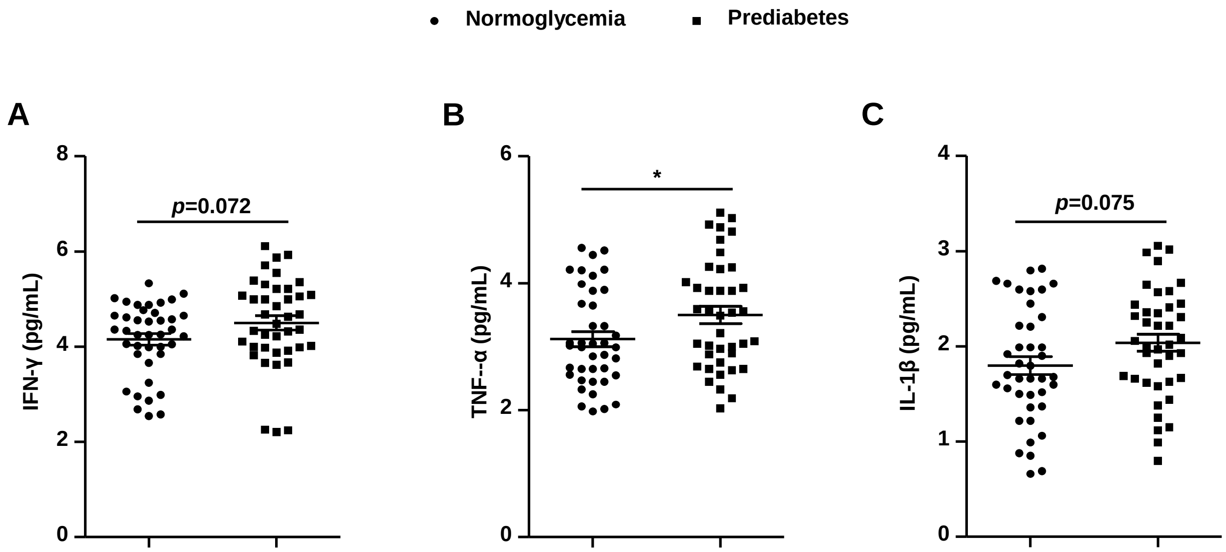

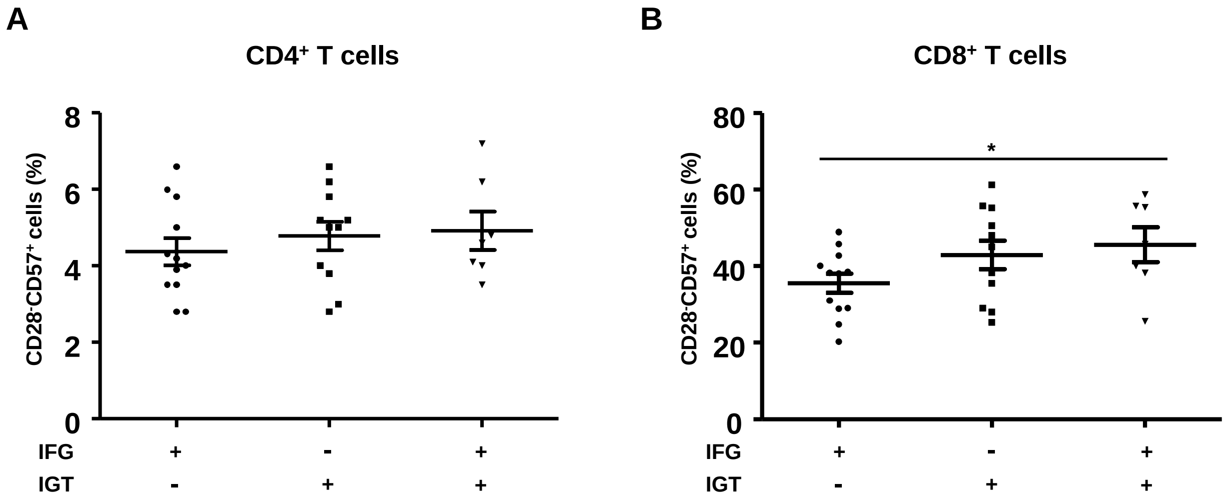
A
CD4+ T cells
CD28-CD57+ cells (%)
8
6
4
2
0
IFG + - +
IGT - + +
B
CD8+ T cells
CD28-CD57+ cells (%)
80
60
40
20
0
*
IFG + - +
IGT - + +

Supplementary Fig. 4

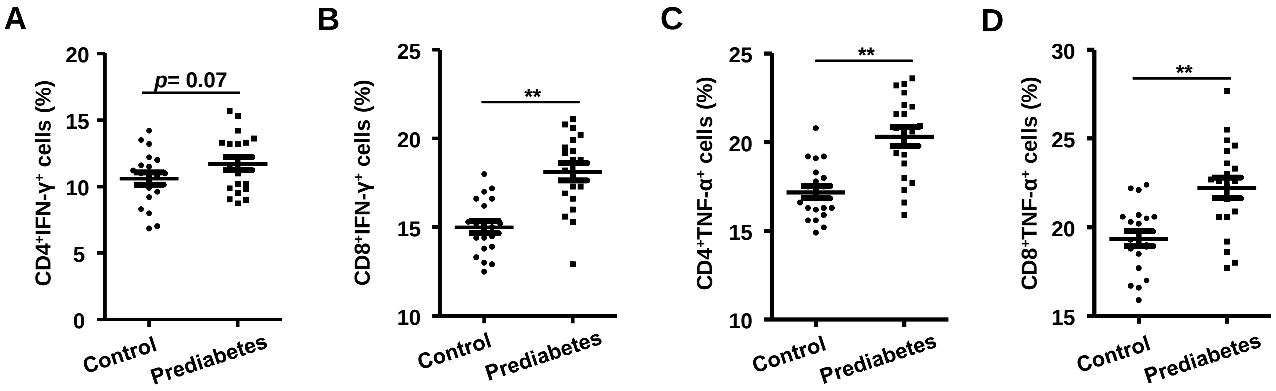

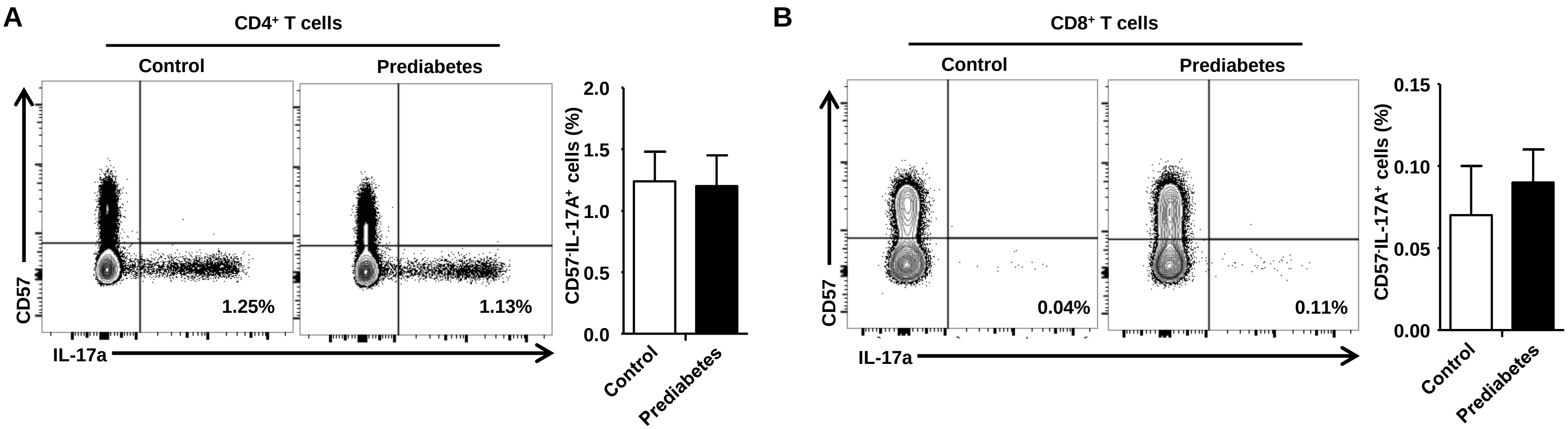
A
CD4+ T cells
Control
Prediabetes
CD57
IL-17a
1.25%
1.13%
CD57-IL-17A+ cells (%)
2.0
1.5
1.0
0.5
0.0
Control
Prediabetes
B
CD8+ T cells
Control
Prediabetes
CD57
IL-17a
0.04%
0.11%
CD57-IL-17A+ cells (%)
0.15
0.10
0.05
0.00
Control
Prediabetes

Supplementary Fig. 6

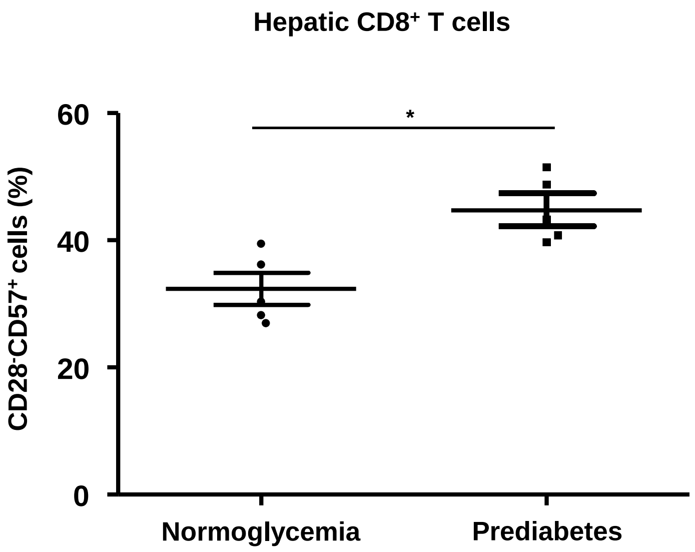

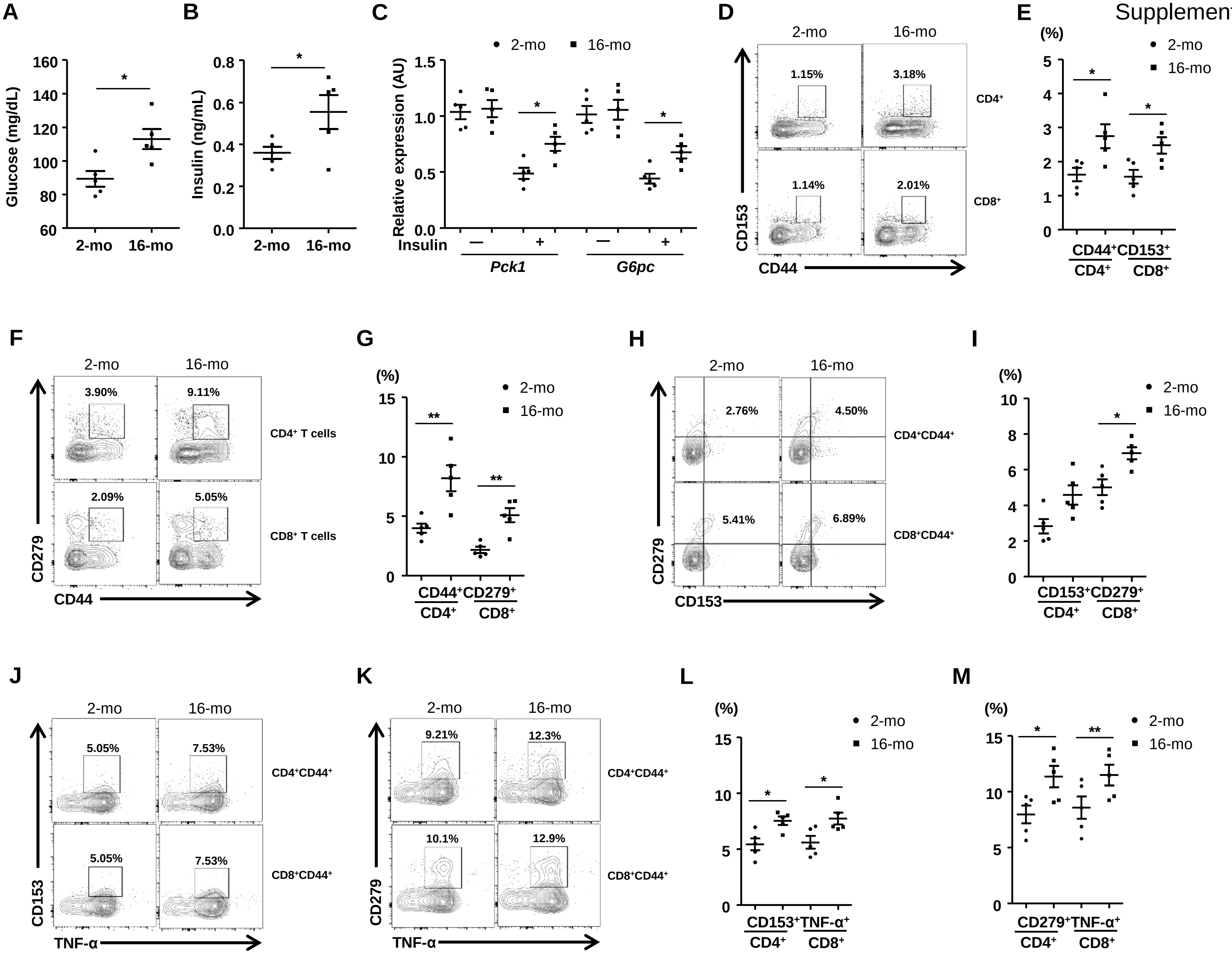

Supplementary Fig. 7

Supplementary Fig. 8

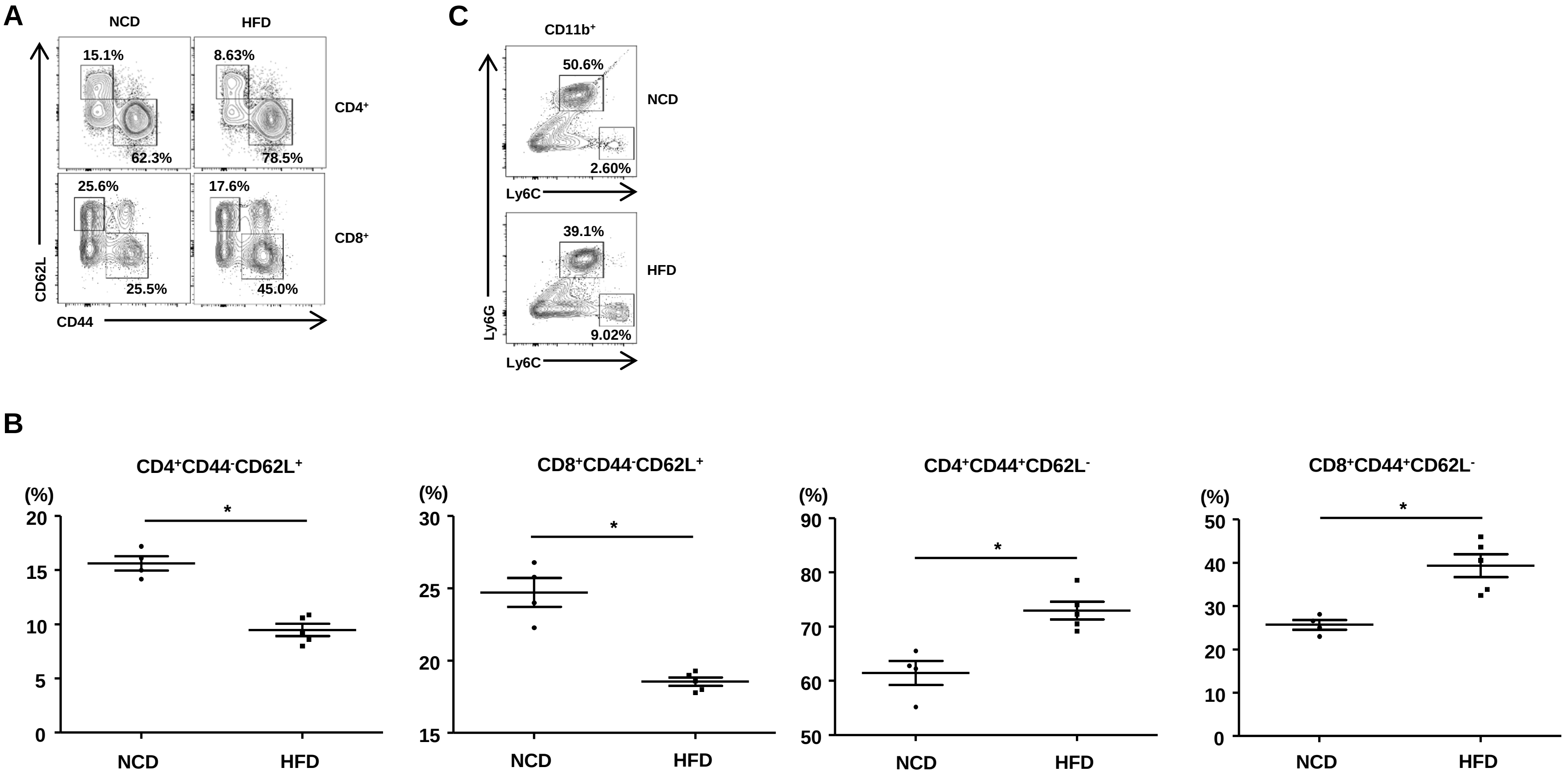

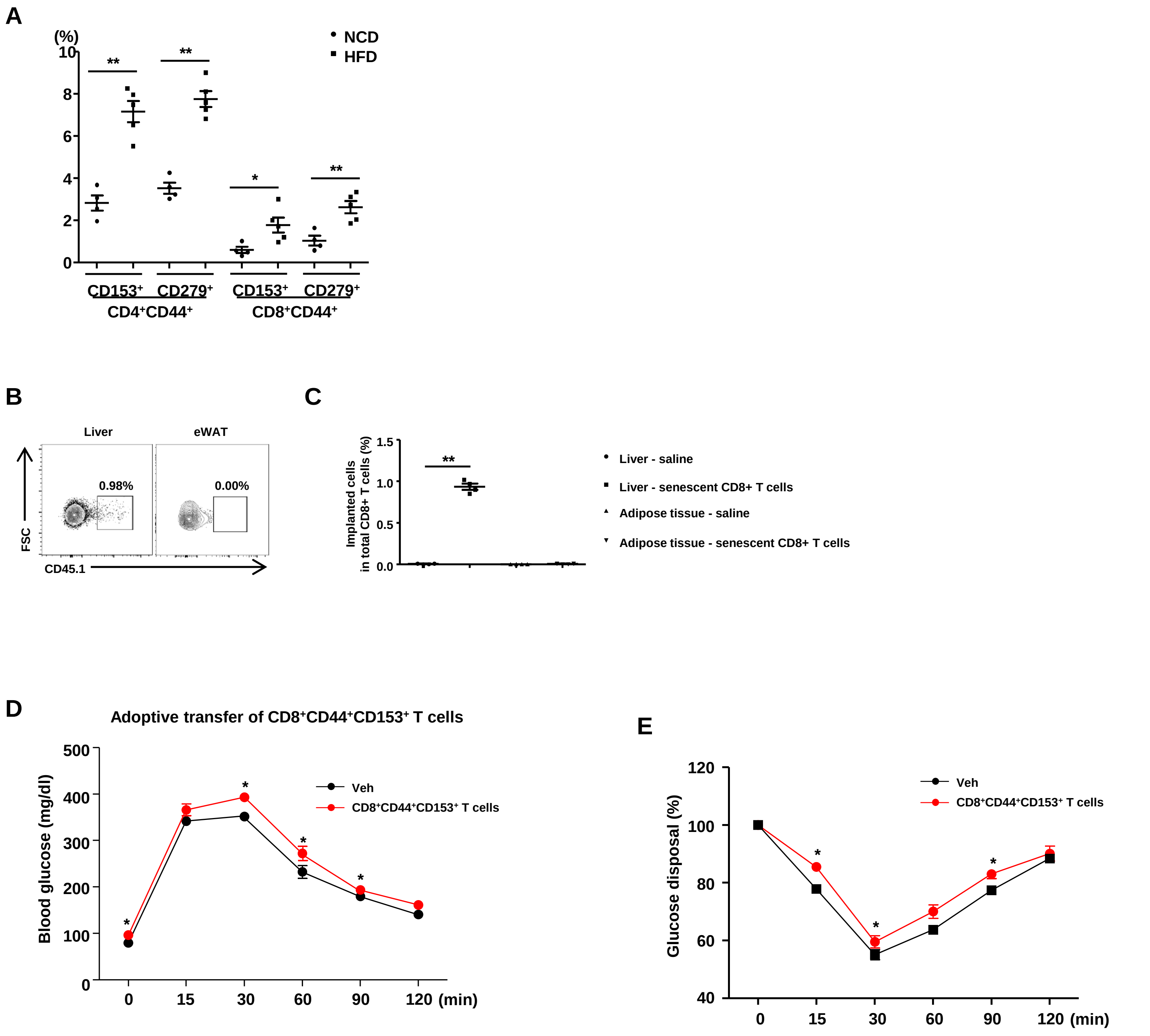
A
(%)
NCD
HFD
**
**
*
**
CD153+
CD279+
CD4+CD44+
CD153+
CD279+
CD8+CD44+
B
Liver
eWAT
0.98%
0.00%
FSC
CD45.1
C
Implanted cells in total CD8+ T cells (%)
**
Liver - saline
Liver - senescent CD8+ T cells
Adipose tissue - saline
Adipose tissue - senescent CD8+ T cells
D
Adoptive transfer of CD8+CD44+CD153+ T cells
Blood glucose (mg/dl)
Veh
CD8+CD44+CD153+ T cells
0 15 30 60 90 120 (min)
E
Glucose disposal (%)
Veh
CD8+CD44+CD153+ T cells
0 15 30 60 90 120 (min)

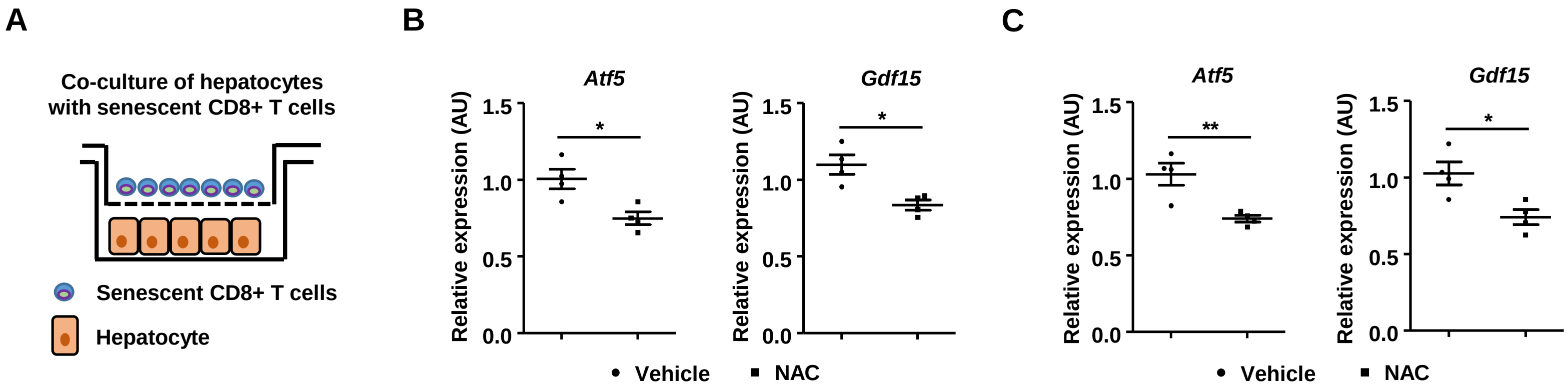
A
Co-culture of hepatocytes with senescent CD8+ T cells
Senescent CD8+ T cells
Hepatocyte
B
Atf5
Gdf15
Relative expression (AU)
1.5
1.0
0.5
0.0
*
Vehicle
NAC
C
Atf5
Gdf15
**
*
