# Supplementary material for: T-cell senescence contributes to abnormal glucose homeostasis in humans and mice
Source: Cell Death Dis. 2019 Mar 13;10(3):249. doi: 10.1038/s41419-019-1494-4 (PMC6416326; doi:10.1038/s41419-019-1494-4)
Supplement: Supplementary file 3 — Supplemental figure legends [file 41419_2019_1494_MOESM3_ESM.docx]

**Supplemental Data**

**T cell senescence contributes to abnormal glucose homeostasis in humans and mice**

- Hyon-Seung Yi^1,2^*^#^, So Yeon Kim^3,4#^, Jung Tae Kim^1,5^, Young-Sun Lee^6^, Ji Sun Moon^1^, Mingyo Kim^7^, Yea Eun Kang^1,2^, Kyong Hye Joung^1,2^, Ju Hee Lee^1,2^, Hyun Jin Kim^1,2^, Kwangsik Chun^8^, Minho Shong^1,2^, Bon Jeong Ku^1,2^*
- ^1^Research Center for Endocrine and Metabolic Diseases, Chungnam National University Hospital, Chungnam National University School of Medicine, Daejeon 35015, Republic of Korea. ^2^Department of Internal Medicine, Chungnam National University School of Medicine, Daejeon 35015, Republic of Korea. ^3^Laboratory of Liver Research, Biomedical Science and Engineering Interdisciplinary Program, Korean Advanced Institute of Science and Technology, Daejeon, 34141, Republic of Korea. ^4^Division of Digestive and Liver Diseases, Department of Medicine Cedars-Sinai Medical Center, Los Angeles, CA 90048. ^5^Department of Medical Science, Chungnam National University School of Medicine, 266 Munhwaro, Daejeon 35015, Republic of Korea. ^6^Department of Internal Medicine, Korea University College of Medicine, Seoul, 08308, Republic of Korea. ^7^Division of Rheumatology, Department of Internal Medicine, Gyeongsang National University School of Medicine, 79, Gangnam-ro, Jinju, Gyeongnam 660-702, Republic of Korea. ^8^Department of Surgery, Chungnam National University School of Medicine, Daejeon 35015, Republic of Korea
- ^#^ These authors contributed equally to this work.

**Legends to supplementary figures**

**Supplementary Figure 1: Identification of differentially expressed genes in PBMCs from normoglycemic controls and patients with prediabetes.** (A-C) Representative bar chart ranking of the top ten Gene Ontology cellular signaling, molecular function, and biological process terms associated with the upregulated genes, based on count. (D) The analysis was performed using Network2Canvas. Genes that were significantly upregulated in the PBMCs of normal controls and patients with prediabetes were analyzed for gene-list enrichment with gene-set libraries created from level 4 of the MGI mouse phenotype ontology using Network2Canvas. Data are expressed as mean ± SEM. ***P* < 0.01, ****P* < 0.001 compared with the corresponding controls.

**Supplementary Figure 2: Levels of serum inflammatory cytokines in normoglycemic subjects and patients with prediabetes.** (A-C) Statistical analysis of the serum levels of IFN-γ, TNF-α and IL-1β in normoglycemic subjects and patients with prediabetes. Data are expressed as mean ± SEM. **P* < 0.05 compared with the corresponding controls.

**Supplementary Figure 3: Population size of CD28^−^CD57^+^ T cells from normoglycemic subjects and patients with prediabetes, according to the diagnostic criteria for prediabetes.** (A-B) Population size of CD28^−^ and CD57^+^ CD4^+^ and CD8^+^ T cells in the four groups, including IFG only, IGT only, and IFG plus IGT. Data are expressed as mean ± SEM. **P* < 0.05, compared with the corresponding controls.

**Supplementary Figure 4: Population size of IFN-γ and TNF-α producing total CD4^+^ and CD8^+^ T cells from normoglycemic subjects and patients with prediabetes.** (A-B) IFN-γ and TNF-α producing total CD4^+^ T cells from normoglycemic subjects and patients with prediabetes. (C-D) IFN-γ and TNF-α producing total CD4^+^ T cells from normoglycemic subjects and patients with prediabetes. Data are expressed as mean ± SEM. **P* < 0.05, ***P* < 0.01 compared with the corresponding controls.

**Supplementary Figure 5: IL-17A-secreting cells in the PBMCs from patients with prediabetes and normoglycemic subjects.**

(A-B) The frequency of IL-17A-secreting cells in the population of CD57^+^ CD4^+^ or CD8^+^ T cells was compared between the two groups. Flow cytometry plots are representative of at least three independent experiments. Data are expressed as mean ± SEM.

**Supplementary Figure 6: Population comparison of human hepatic CD8+ senescent T cells between patients with prediabetes and subjects with normoglycemia.** Data are expressed as mean ± SEM. **P* < 0.05 compared with the corresponding controls.

**Supplementary Figure 7: CD44^+^CD153^+^ and CD44^+^CD279^+^ subsets of CD4^+^ and CD8^+^ T cells are present in greater numbers in the livers of aged mice.** (A-B) Analysis of fasting blood glucose and insulin in young (2-month-old; *n*=5) and old (16-month-old; *n*=5) mice. (C) Primary hepatocytes isolated from young and old mice were treated with insulin (100 nM) or saline for 4 h, and then subjected to PCR analysis. (D-E) FACS analysis of hepatic CD4^+^CD44^+^CD153^+^ and CD8^+^CD44^+^CD153^+^ T cells isolated from young (*n*=5) and old mice (*n*=5). (F-G) FACS analysis of hepatic CD4^+^CD44^+^CD279^+^ and CD8^+^CD44^+^CD279^+^ T cells isolated from young (*n*=5) and old mice (*n*=5). (H-I) FACS analysis of CD4^+^CD44^+^CD153^+^CD279^+^ and CD8^+^CD44^+^CD153^+^CD279^+^ T cells isolated from the liver of young (2-month-old; *n*=5) and old (16-month-old; *n*=5) mice. (J-M) TNF-α expression of hepatic CD4^+^CD44^+^CD153^+^, CD4^+^CD44^+^CD279^+^, CD8^+^CD44^+^CD153^+^ and CD8^+^CD44^+^CD279^+^ T cells isolated from young (*n*=5) and old mice (*n*=5). Data are expressed as mean ± SEM. **P* < 0.05, ***P* < 0.01 compared with the corresponding controls. Flow cytometry plots are representative of at least three independent experiments.

**Supplementary Figure 8: CD44^+^ T cells are more numerous in the livers of mice fed high fat diet.** (A, B) Hepatic CD44^+^CD62^-^ and CD44^-^CD62^+^ subsets of CD4^+^ and CD8^+^ T cells from mice fed a NCD (*n*=4) or a HFD (*n*=5). (C) Hepatic infiltrating monocytes and neutrophils from mice fed a NCD or a HFD. Data are expressed as mean ± SEM. **P* < 0.05 compared with the corresponding controls.

**Supplementary Figure 9: Mice fed a high fat diet exhibit an increase of senescent T cells, which produce glucose intolerance and insulin resistance in mice.** (A) FACS analysis of hepatic CD4^+^CD44^+^CD153^+^, CD8^+^CD44^+^CD153^+^, CD4^+^CD44^+^CD279^+^, and CD8^+^CD44^+^CD279^+^ T cells isolated from mice fed a NCD (*n*=4) or a HFD (*n*=5). (B, C) FACS analysis for hepatic and adipose population of adoptive transferred CD8^+^CD44^+^CD153^+^ T cells from CD45.1 mice to CD45.2 mice. (D, E) Effects of adoptive transfer of CD8^+^CD44^+^CD153^+^ T cells from mice fed a NCD as determined by glucose tolerance test and insulin tolerance test. Data are expressed as mean ± SEM. **P* < 0.05, ***P* < 0.01 compared with the corresponding controls.

**Supplementary Figure 10: Co-culturing murine hepatocytes and senescent CD8^+^ T cells in *in vitro*.** (A) Schematic model of co-culturing hepatocytes and senescent CD8^+^ T cells using Transwell insert. (B) Real-time PCR analysis of primary hepatocytes co-cultured with CD8^+^CD44^+^CD153^+^ T cells with or without N-acetylcysteine (5 mM) for 6 hours. (C) Real-time PCR analysis of primary hepatocytes treated with conditioned media of hepatic senescent CD8^+^ T cells treated with or without N-acetylcysteine (5 mM) for 6 hours. Data are expressed as mean ± SEM. **P* < 0.05, ***P* < 0.01 compared with the corresponding controls.

**Supplementary Table 1: Clinical characteristics of human liver tissue donors.**

| Group | No | Gender | Age | Body mass index | FBS | Diagnosis | Liver metastasis |
| --- | --- | --- | --- | --- | --- | --- | --- |
| Normoglycemia | 1 | M | 52 | 21.23 | 99 | HCC | N/A |
|  | 2 | F | 49 | 27.2 | 87 | HCC | N/A |
|  | 3 | M | 50 | 18.1 | 88 | HCC | N/A |
|  | 4 | F | 52 | 23.8 | 91 | Colon cancer | Y |
|  | 5 | M | 61 | 21.6 | 96 | HCC | N/A |
| Diabetes | 1 | M | 48 | 20.6 | 192 | HCC | N/A |
|  | 2 | M | 61 | 20.1 | 201 | HCC | N/A |
|  | 3 | M | 60 | 25 | 145 | HCC | N/A |
|  | 4 | M | 85 | 24.1 | 168 | HCC | N/A |
|  | 5 | M | 53 | 22.3 | 144 | HCC | N/A |

BMI, body mass index; FBS, fasting blood sugar; HCC, hepatocellular carcinoma; N/A, not applicable.

**Supplementary Table 2: Primers used in real-time PCR (human)**

| Genes | Forward (5’-3’) | Reverse (5’-3’) | PCR product (base pairs) |
| --- | --- | --- | --- |
| *CD44* | CTGCCGCTTTGCAGGTGTA | CATTGTGGGCAAGGTGCTATT | 109 |
| *CD69* | ATTGTCCAGGCCAATACACATT | CCTCTCTACCTGCGTATCGTTTT | 219 |
| *CD83* | AAGGGGCAAAATGGTTCTTTCG | GCACCTGTATGTCCCCGAG | 96 |
| *IFNG* | TCGGTAACTGACTTGAATGTCCA | TCGCTTCCCTGTTTTAGCTGC | 93 |
| *TNF* | CCTCTCTCTAATCAGCCCTCTG | GAGGACCTGGGAGTAGATGAG | 220 |
| *IL1B* | TTCGACACATGGGATAACGAGG | TTTTTGCTGTGAGTCCCGGAG | 84 |
| *IL17A* | AGATTACTACAACCGATCCACCT | GGGGACAGAGTTCATGTGGTA | 151 |
| *G6PC* | CTACTACAGCAACACTTCCGTG | GGTCGGCTTTATCTTTCCCTGA | 160 |
| *PCK1* | TTGAGAAAGCGTTCAATGCCA | CACGTAGGGTGAATCCGTCAG | 134 |
| *ACTB* | CATGTACGTTGCTATCCAGGC | CTCCTTAATGTCACGCACGAT | 250 |
| *GADPH* | CTGGGCTACACTGAGCACC | AAGTGGTCGTTGAGGGCAATG | 101 |
